# Dynamic Covalent Chemistry-based Sensing: Pyrenyl Derivatives of Phenylboronic Acid for Saccharide and Formaldehyde

**DOI:** 10.1038/srep31187

**Published:** 2016-08-08

**Authors:** Xingmao Chang, Jiayun Fan, Min Wang, Zhaolong Wang, Haonan Peng, Gang He, Yu Fang

**Affiliations:** 1 Key Laboratory of Applied Surface and Colloid Chemistry (Ministry of Education), Shaanxi Normal University, School of Materials Science and Engineering, Xi’an, 710119, P. R. China; 2 Key Laboratory of Applied Surface and Colloid Chemistry (Ministry of Education), Shaanxi Normal University, School of Chemistry and Chemical Engineering, Xi’an, 710119, P. R. China; 3 Frontier Institute of Science and Technology, Xi’an Jiaotong University, Xi’an 710054, P. R. China

## Abstract

We synthesized two specially designed pyrenyl (Py) derivatives of phenylboronic acid, PSNB1 and PSNB2, of which PSNB2 self-assemble to form dynamic aggregate in methanol-water mixture (1:99, v/v) via intermolecular H-bonding and pi-pi stacking. Interestingly, the dynamic aggregate shows smart response to presence of fructose (F) as evidenced by fluorescence color change from green to blue. More interestingly, the fluorescence emission of the resulted PSNB2-F changes from blue to green with the addition of formaldehyde (FA). The reason behind is formation of a PSNB2-F dimer via FA cross-linking. Based upon the reactions as found, sensitive and fast sensing of F and FA in water was realized, of which the experimental DLs could be significantly lower than 10 μM for both analytes, and the response times are less than 1 min. It is believed that not only the materials as created may have the potential to find real-life applications but also the strategy as developed can be adopted to develop other dynamic materials.

Supramolecular and dynamic polymers are two kinds of non-conventional polymers, and they are composed of small structural units, of which any size could be connected by various non-covalent interactions for the former like hydrogenbonding^1^,^2^, metal-coordination^3^,^4^, π-π interaction^5^,^6^,^7^, and host-guest interaction^8^,^9^,^10^,^11^,^12^,^13^, but for the later, however, like dynamic covalent interaction, electron or proton transfer-based ionic association, etc.^14^,^15^,^16^. These polymers are unique because the non-covalent or dynamic covalent linkage may impart dynamic, reversible, and degradable characteristics tothem, and thereby could be employed as general principles for design of sensing materials^17^,^18^,^19^,^20^,^21^,^22^.

As it is well known, boronic acids could combine cis-1,2-diolmoieties of saccharides rapidly and reversibly via formation of cyclic boronate esters^23^. Based on this specific reaction, a variety of synthetic chemosensors for saccharides have been created^24^,^25^,^26^,^27^,^28^,^29^,^30^,^31^,^32^. The signals recorded or observed from the sensors are generally fluorescence, and the principles behind are intramolecular charger transfer (ICT) or photo-induced electron transfer (PET)^33^,^34^,^35^. The sensitivity and selectivity of the detection has been greatly improved recently due to employment of self-assembled structures even though the principle is still formation of conventional cyclic boronate esters^36^,^37^,^38^,^39^,^40^.

Formaldehyde (FA) is a highly reactive carbonyl species produced in living systems that has been implicated in the pathologies of various cancers, diabetes, heart, liver and neurodegenerative diseases^41^,^42^,^43^. Despite its significance, convenient and rapid monitoring of FA in solution and in living systems remains a big challenge. Conventional methods for FA detection based on colorimetric estimation^44^, radiometry^45^, gas chromatography^46^, selected ion flow tube mass spectrometry^47^, and high-performance liquid chromatography^48^, may offer high sensitivity and good selectivity but they are limited by tedious pretreatment of samples and/or require invasive destruction of biological tissues. Recently, two sensitive and selective fluorescent sensors for FA detection in solution and living systems were reported^49^,^50^. However, equilibration of the sensors’ response needs a few hours. Therefore, fast detection of FA still remains a challenge. Moreover, presence of saccharides within cells is inevitable, and thereby in the detection, interference from them must be taken into account. In other words, simultaneous detection of saccharides and FA with a single fluorophore can be beneficial for both detections. However, to our knowledge, this effort has never been reported before. This paper reports a successful effort in this regard, which was realized via utilization of a balance between aggregation and disaggregation of a specially designed pyrenyl derivative of phenylboronic acid and dimerization of the compound via FA cross-linking^51^. The assembly and disassembly of the dynamic aggregates, as well as the formation of the dimer are schematically shown in Fig. 1. Clearly, the most pronounced feature of the response of the fluorescent compound to the chemicals as focused is its change in molecular structure before and after the recognition event, a typical dynamic chemistry process^52^,^53^,^54^.

To have a better understanding of the recognition, two pyrenyl derivatives bearing boronic acid and secondary amine structures, PSNB1 and PSNB2, were designed and synthesized. The structures and synthesis routes of them are depicted in Scheme S1 (Supporting information). It is seen that the difference between the two compounds is only on the relative positions of the boronic acid group and the imino structure on the phenylring. In this paper, we report on the results from the studies of the dynamic aggregate formation of PSNB2 and its utilization for the sensitive and fast sensing of fructose (F). In addition, the results from the studies of the interaction between PSNB2-F and FA will also be presented.

## Results and Discussion

### Solution behavior of PSNB1 and PSNB2

#### Concentration effect

**Figure 1:**
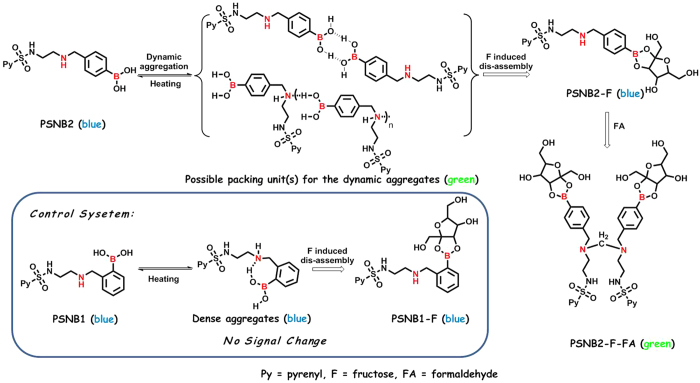
The structures of PSNB1 and PSNB2, as well as the schematic representation of the formation and transformation of PSNB2 relevant dynamic aggregate and molecular dimer. Note: To be clear, the color of each state is denoted.

**Figure 2:**
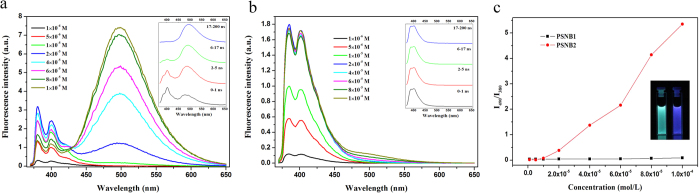
Fluorescence emission spectra of PSNB2 (**a**) and PSNB1 (**b**) in carbonate buffer (pH = 9.0) at different concentrations, respectively. Insets of Fig. 2a,b are time-resolved emission spectra of PSNB2 and PSNB1, respectively, which were recorded in carbonate buffer using pico-second pulsed diode laser (EPL-343.3) as an excitation source (pH = 9.0/1.0 × 10^−4^ mol·L^−1^). (**c**) Plots of I_490_/I_380_ against the concentration of PSNB1 and PSNB2. The inset is pictures of PSNB2 (left) and PSNB1 (right) in the buffer at a concentration of 1.0 × 10^−4^ mol·L^−1^.

As shown in Fig. 1, it was expected that PSNB1 and PSNB2 should show different solution behavior due to its difference on the structure. To interrogate the differences, concentration effect upon the solution behavior of the two compounds was conducted by monitoring their fluorescence changes. The results are depicted in Fig. 2a,c.

With reference to Fig. 2b, it is seen that the profile of the fluorescence emission of PSNB1 is almost concentration independent except those of the systems with higher concentrations, of which small but obvious Py excimer emissions could be observed. For the systems of PSNB2 (c.f. Fig. 2a), however, the profile of the emission is very much concentration dependent. It is seen that Py excimer starts to form at a concentration of 1.0 × 10^−5^ mol/L, and the relative intensity of the emission from the excimer increases along with increasing the compound concentration. Figure 2c depicts plots of the ratio of I_490_/I_380_, of which I_490_ stands for the intensity of the Py excimer emission and I_380_ the monomer emission of the same fluorophore, respectively, against each of the compounds concentration. As discussed already, for PSNB1, the ratio almost keeps constant within the whole concentration range studied, but for PSNB2, the ratio increases along with increasing the compound concentration, and eventually exceeds 5 when the concentration reaches 1.0 × 10^−4^ mol/L, indicating directly that the two compounds behavior differently in the solutions. The observation may be rationalized by considering the specific structures of them. For PSNB2, the relative positions of the boronic acid group and the imino structure on the benzene ring may prohibit their intra-molecular interaction such as hydrogen bonding between the two functional groups, but favor inter-molecular bonding such as interactions between different boronic acids and/or hydrogen bonding between the imino structure of a PSNB2 molecule and the boronic acid structure of another PSNB2 molecule, and thereby result in aggregation of the compound in the solution, promoting Py excimer formation. For PSNB1, however, the priority in the formation of the intra-molecular hydrogen bond may prohibit the association of the compound and screen Py excimer formation, which explains why there was no significant Py excimer emission from the solutions of this compound even at much higher concentrations. The difference in the discussed interactions are schematically shown in Fig. 1. This tentative elaboration is further supported by the results from TRES and other additional studies.

#### Time-resolved emission spectroscopy study

Time-resolved emission spectroscopy (TRES) is a powerful technique to interrogate the behavior of a fluorophore at its excited state. To have a deeper understanding of the solution behavior of PSNB2, TRES measurements were conducted for the system of PSNB2/methanol:water (1:99, v/v, 1 × 10^−4^ M). The results are depicted in the inset of Fig. 2a. With reference to the spectra, it is revealed that the profile of the earliest time gate (0–1 ns) spectrum is characterized by Py monomer emission (~400 nm) and distorted Py excimer emission around 480 nm. With the time gate moving to longer time (2–5 ns and 6–17 ns), the structure of the emission changes as evidenced by the fact that the excimer emission starts to dominate and shifts to longer wavelengths. For the latest time gate (17–200 ns) spectrum, the Py monomer emission almost disappears. These results suggest that the Py excimers might be mainly formed via a pre-formed scheme that is direct excitation of the ground state dimers of the Py unit^55^, as depicted in E.g. 1.


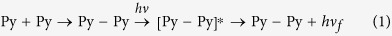


It is to be noted that the basic requirement for ground state dimer formation is that the two relevant units must come into contact with each other, a strong evidence of aggregation of the molecules of PSNB2 within the solution under study. In contrast, for the system of PSNB1, such phenomenon was not observed, a result understandable by considering the elaborations described in section 2.1.1(c. f. Inset of Figs 2b and 1).

### Aggregation of PSNB2

#### Diffusion ordered spectroscopy (DOSY)

Initially, formation of dynamic polymers of PSNB2 was expected owing to its inter-molecular interaction. To confirm the presence of the polymer, diffusion ordered spectroscopy (DOSY)^20^,^56^ measurements of PSNB2 and the reference PSNB1 in DMSO-d6 were conducted. To have a better understanding of the difference between the two compounds in solution, they were firstly protonated with deuterated hydrochloride (DCl) to allow them to be freed from the possible dynamic polymers or aggregates. In this way, the diffusion coefficients of them (PSNB1 + DCl and PSNB2 + DCl) in DMSO-d6 were measured and the results are 1.17 × 10^−10^ m^2^s^−1^ and 1.13 × 10^−10^ m^2^s^−1^, respectively (c.f. Fig. S1), suggesting that the sizes of the two compounds in protonated state are close to each other. However, in un-protonated state, the diffusion coefficient of PSNB1 is larger than that of PSNB2, indicating formation of larger aggregates, which may be taken as an evidence for the formation of the expected dynamic aggregates/polymers via possible intermolecular hydrogen bonding and/or boronic acid-boronic acid interaction. To further confirm this tentative result, a control compound PSNP, which has similar size and structure with PSNB but possesses no boronic acid group, was utilized to conduct the same diffusion test, and the result is also shown in the Figure. It is seen that the diffusion coefficient of this reference compound is 1.66 × 10^−10^ m^2^s^−1^, very close to the value of PSNB1 (1.65 × 10^−10^ m^2^s^−1^) but more than 16.7% larger than that of PSNB2 (1.37 × 10^−10^ m^2^s^−1^), confirming the association of PSNB2 in the solution tested. However, it is to be noted that the protonated species of both PSNB1 and PSNB2 show much lower diffusion coefficients than the corresponding neutral compounds and the control compound, PSNP, this can be only rationalized by considering that the three compounds under study are all existing in aggregated states, and the only difference is that the sizes of the aggregates may be different. In other words, PSNB2 exhibits much stronger tendency to form aggregates, which explains why PSNB2 shows more pronounced excimer emission in solution state.

As for why PSNB1 shows little excimer emission in the similar solution even though aggregation is also observed, the reason might be that the aggregates formed by this compound are mainly composed of monomeric PSNB1 because it has no chance to form dynamic polymers due to the reasons afore elaborated, and thereby densely packed aggregates might be formed, which is unfavorable for the formation of Py excimers. This tentative explanation is confirmed by the appearance of the sharp peaks in the XRD traces of the PSNB1 aggregates (c.f. Fig. S2). In contrast, for PSNB2, it possesses the chance to form dynamic polymers due to specific inter-molecular interactions, and polymerization of this compound may prevent formation of dense and ordered aggregates as confirmed by disappearance of the sharp peaks in the corresponding XRD traces (c.f. Fig. S2). Formation of the random and loosely packed aggregates must favor formation of Py excimers due to greater mobility of the pyrenyl units within the aggregates.

#### ^1^H NMR measurements

^1^H NMR studies were conducted to obtain further information about the formation of the PSNB2-based dynamic aggregates. Figure S3a shows the temperature dependent ^1^H NMR spectra of the compound in DMSO-d_6_, of which the concentration of the compound in the solvent is 1.6% (*w/v*). With reference to the spectra shown in the Figure, it is seen that the hydroxyl signal from boronic acid shifted from 7.94 ppm at 298 K to 7.70 ppm at 338 K. The NH signal from the sulfonamide structure shifted from 5.75 to 5.70 ppm over the same temperature range. The changes in the signal positions could be ascribed to the weakening of the inter- and/or intra-molecular hydrogen-bonding interaction due to increase in the temperatureof the solution^57^.

To make clear inter- or intra-molecular nature of the interaction, additional concentration-dependent ^1^H NMR measurements were further conducted, and the results are shown in Fig. S3b. Reference to the spectra depicted in the Figure reveals that the relative contribution of intermolecular H-bonding interactions also increases. In the range of 0.2% (~0.02 mol/L) to 1.6% (0.14 mol/L) PSNB2, signals of the hydroxyl from boronic acids and NH group shifted to lower field with increasing concentration. The pyrenyl signals provide additional information about π-π stacking. The chemical shift of the α-H of the pyrenyl ring in PSNB2 (1.6%) appears at 8.99–8.97 ppm when the measurement was conducted at 298 K, and the signal moves to 9.03–9.01 ppm at 338 K (c.f. Fig. S3a) which affords noticeable evidence for the existence of π-π stacked arrangement of the pyrenyl moieties in the dynamic aggregates. ^1^H NMR studies indicated that the intermolecular H-bonding interactions plays key role in the formation of the dynamic aggregates and π-π stacked interactions of pyrenyl moieties are likely to be another driving force for the aggregation of the compound, a conclusion which will be further confirmed by Transmission Electron Microscopy (TEM) and Dynamic Light Scattering (DLS) studies.

#### Fluorescence studies

**Figure 3:**
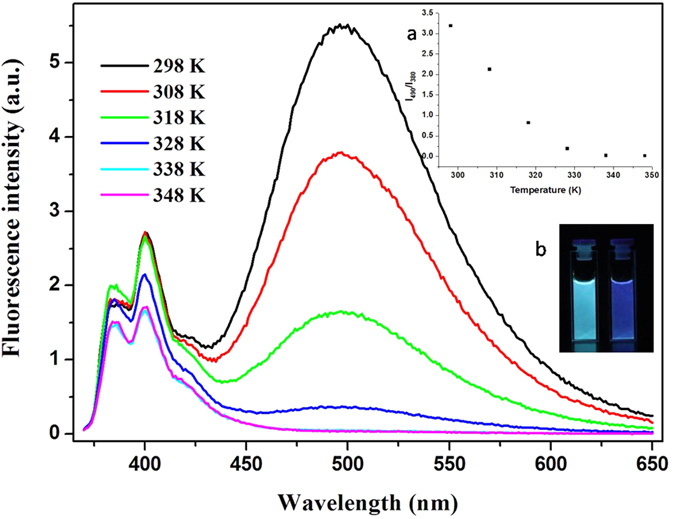
Temperature-dependent fluorescence emission spectra of PSNB2 (pH = 9.0/1.0 × 10^−4^ mol·L^−1^). The inset a is a plot of I_490_/I_380_ against the temperature, and inset b is a picture of PSNB2 solution taken before (left) and after (right) heating under UV light.

It can be anticipated that the efficiency of Py excimer emission in the PSNB2 solution under study is temperature dependence because formation of them is a reversible process. Accordingly, temperature effect on the excimer formation of the dynamic system was examined and the results are shown in Fig. 3. Reference to the Figure reveals that the profiles of the fluorescence emissions are largely dependent upon the temperature. Specifically, along with temperature increase from 298 K to 348 K, the excimer emission around 495 nm decreased dramatically, but the monomer emission between 360 and 430 nm was kept almost un-changed, resulting in decrease of I_490_/I_380_ which is accompanied by fluorescence color change from green to blue (c.f. inset a and b of Fig. 3). The phenomena observed from the system may be explained by considering that increase in temperature must enhance degradation of the dynamic aggregates under discussion, and the degradation will definitely reduce excimer emission, which must be reflected by decrease in the value of I_490_/I_380_.

As for the observation that decrease in the excimer emission does not result in increase in the monomer emission, it may be understood by considering the competition between radiative and non-radiative deactivation. It is known that increase in temperature will increase the probability of non-radiative decay of the excited state of a fluorophore due to enhanced collision of the fluorophore with the solvent molecules around, and this increase must inhibit the radiative decay, resulting in the phenomenon as observed. The properties as revealed demonstrate the dynamic nature of the systems under study, which is crucial for them to find important applications.

#### TEM and DLS studies

**Figure 4:**
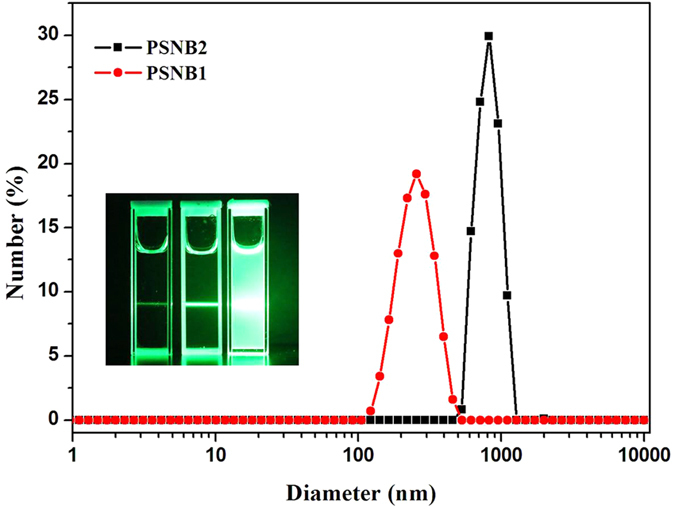
Particle size distributions of PSNB1 and PSNB2 dissolved in aqueous solution (1.0 × 10^−4^ mol/L). Inset picture shows the Tyndall scattering result of samples (from left to right, buffer, PSNB1, and PSNB2).

TEM and DLS experiments were used for the studies of the aggregation behavior of PSNB2 in solution state. TEM measurements revealed that PSNB2 aggregated into spherical particles, of which the diameters vary from 500 to 600 nm, at a concentration of 1 × 10^−4^ mol/L (c.f. Fig. S4). As TEM measurements were conducted after evaporation of the solvent, the aggregates shown in the TEM pictures could be formed during the evaporation process. To further examine the aggregation of the compound in the system, DLS measurements were also performed. Figure 4 depicts the result from the studies. Reference to the Figure demonstrates that the average diameter of the aggregates is about 800 nm, but for PSNB1, the diameters are much smaller even though it also forms aggregates. Clearly, the average size of the aggregates presenting in the PSNB2 system recorded from TEM measurements is significantly smaller than that obtained from DLS studies. The difference between the two measurements could be rationalized by considering the fact that DLS measurements were conducted in solution state that means that the aggregates are in swelled state, but for TEM measurements that aggregates are in dried state^58^. Presence of the aggregates was further confirmed by Tynda II effect as shown in the inset of Fig. 4. Further inspection of the scattering reveals that aggregation of PSNB2 in its solution is more obvious than that of PSNB1 as evidenced by much more obvious scattering. These results suggest that the strong excimer emission of pyrene in the PSNB2 solution must be a result of packing of the pyrenyl units in the aggregates. But for PSNB1, aggregation may not result in efficient packing of the pyrenyl units as there is no significant excimer emission from the system.

### Sensing performance studies

#### Detection of saccharides

It would be no difficult to find that the dynamic nature of the PSNB2-based aggregates may make it a possible chemical sensor for some specific analytes such as saccharides through the dis-assembly of the pyrenyl packings. This is because the dis-assembly must be accompanied by decrease in the ratio of Py excimer emission to Py monomer emission. Considering the importance of saccharide sensing, and the unique sensing principle as described, it was decided to look at the sensing performance of the dynamic aggregates. To conduct the test, fructose was chosen as an example saccharide.

**Figure 5:**
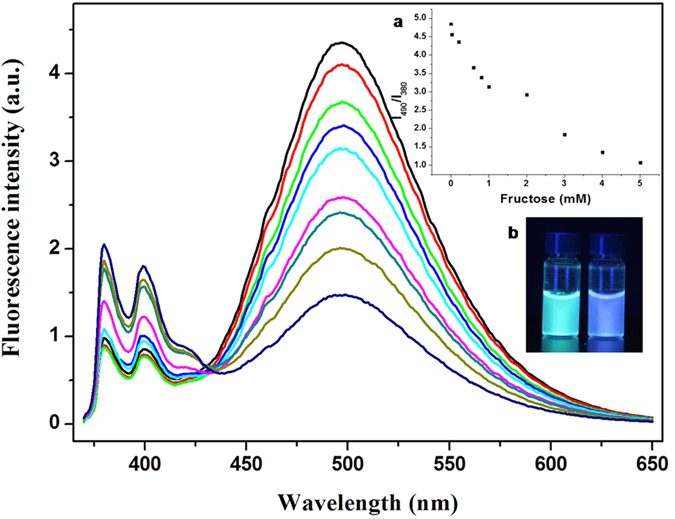
Fluorescence emission spectra of PSNB2 in pH 9.0 carbonate buffer containing 1% (v/v) methanol in the presence of fructose over 0–5 mM. The inserted figures are the plot of I_490_/I_380_ against the concentration of fructose (**a**) and the picture of PSNB2 in the buffer in absence (left) and presence (right) of 5 mM fructose (**b**).

As revealed by others, the stability of boronic acid esters is pH dependent^59^. Therefore, fructose was added to the PSNB2 solution at different pHs, and the corresponding fluorescence emission as well as that of the blank solution were recorded. The values of I_490_/I_380_ from the measurements were plotted as functions of pH (cf. Fig. S5). Examination of the results reveals that pHs around 9 are more suitable than others due to larger difference between the value of I_490_/I_380_ of the sample system and that of the blank one. Figure 5 manifests the fluorescence emission spectra of the systems in the presence of different concentrations of fructose. With reference to the inset of Fig. 5, it is seen that the excimer emission decreases and the monomer emission increases along with increasing the saccharide concentration, a result understandable by considering the afore-mentioned disruption of the dynamic aggregates. For the system of PSNB1, however, addition of fructose results in little change in the fluorescence emission of the system, again, a result understandable by considering the original structure of the compound in solution (Fig. S6). The inset b of Fig. 5 demonstrates that the saccharide sensing could also be realized via a visualization manner.

To further confirm the disruption of the aggregates, DLS tests were also conducted. Figure S7 shows the fructose concentration dependence of the average diameters of the PSNB2 aggregates in the solutions at pH 9. Clearly, the average size of the aggregates decreased from 820 nm to 285 nm with the concentration of fructose increased from 0 mM to 5 mM, and then to 220 nm when the saccharide concentration further increased to 10 mM, a direct evidence for the breakage of the aggregates. It is to be noted that with this strategy a detection limit of 10 μM for fructose could be reached with no difficult, and furthermore the sensing is almost instantaneous (c.f. Fig. S8).

#### Detection of FA

**Figure 6:**
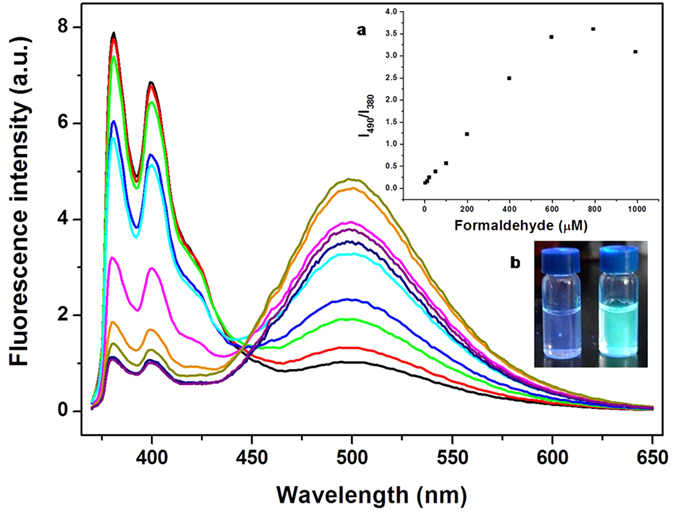
Fluorescence emission spectra of PSNB2-F ([PSNB2] 0.1 mM/[Fructose] 5 mM) in PBS buffer (pH 7.4) in the presence of FA over 0–1 mM. The inserted figures are the plot of I_490_/I_380_ against the concentration of FA (**a**) and the picture of PSNB2-F in the buffer in absence (left) and presence (right) of 1 mM FA (**b**).

Considering the reactivity of FA with imino group and presence of a free imino structure in PSNB2, it was expected that the fluorescence emission of the aqueous solution of the compound would be sensitive to the presence of FA. In this study, the adduct of PSNB2 with fructose (PSNB2-F) was employed for the test of FA instead of PSNB2 because combination of saccharide improves the solubility of the fluorescent compound in aqueous phase. Figure 6 shows the fluorescence emission spectra of PSNB2-F (0.1 mM/10 mM) dissolved in a buffer solution (pH 7.4) in the presence of different concentrations of FA. It is seen that in the absence of FA, the spectrum is dominated by Py monomer emission. However, with increasing FA concentration, the monomer emission decreases and the excimer emission centering around 490 nm increases. As shown in inset b of the Figure, the change can be observed by naked eyes under illumination of UV light. Further analysis of the spectra change could result in a plot shown in inset a of Fig. 6, which manifests that with increasing FA concentration from 0 to 0.8 mM, the ratio of I_490_/I_380_ increased from 0.13 to 3.61, corresponding to 28 times signal change. With further inspection of the spectra, it is seen that the method developed for FA detection in aqueous phase is much more sensitive than the results reported in literatures as presence of 10 μM FA could result in a significant change in the profile of the fluorescence emission of the system. Moreover, the method is superior to others as it possesses an unprecedented response speed, of which introduction of FA is accompanied by almost instant fluorescence change (c.f. Fig. S9).

FA is important for human health, and in the brains of healthy individuals, the concentration of FA lies in the range between 0.2 and 0.4 mM^43^, which implies that the method developed in this work has the potential to be used for FA detection in biological systems because of its good compatibility with water, excellent sensitivity and fast response speed.

To verify if the observed fluorescence change is really originating from the reactions proposed (c.f. Fig. 1). Additional experiments were conducted. First, the adduct of PSNB2-F with fructose, PSNB2-F-FA, was directly observed in the result of MS measurement, of which the sample under test was obtained by evaporating the mixture solution of PSNB2-F and FA (c.f. Fig. S10a–10d). Second, ^1^H NMR test of a model system containing diethylamine (DEA) and FA confirmed the presence of the expected reaction (c.f. Fig. S11). The details of the analysis of the MS and ^1^H NMR measurements are provided in the supporting information.

**Figure 7:**
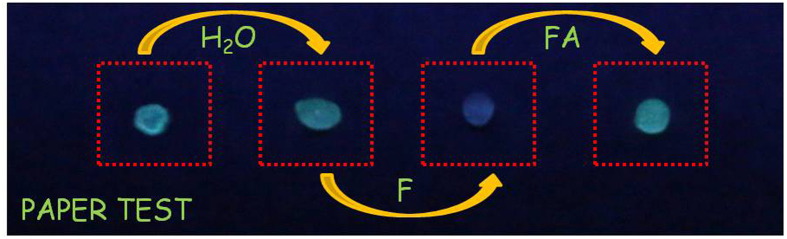
Paper test based on the dynamic aggregates. The picture of PSNB2 (10 mM/methanol, 15 μL) coated on the paper, successively add 20 μL water, 10 μL fructose (1 mM) and 10 μL formaldehyde (0.1 mM) from left to right.

No doubt, it would be more interesting if the sensing tests as described could be conducted in a visualized manner. To verify the feasibility of the tests, a common filter paper was dipped in an aqueous solution of PSNB2, and then dried in air. The paper as obtained was used as a test paper for saccharides. Addition of a small drop of fructose solution could result in significant fluorescent color change. Further introduction of HCHO reversed the change as expected (c.f. Fig. 7).

## Conclusion

Inspired by the concept of dynamic covalent chemistry, two fluorescent compounds, PSNB1 and PSNB2, of which each of them possesses both a secondary amine group and a boronic acid structure were designed and synthesized. Fluorescence studies revealed that PSNB2 shows Py excimer emission but PSNB1 does not. The observation was rationalized by assuming dynamic aggregation of PSNB2 due to intermolecular hydrogen bonding and pi-pi stacking. For PSNB1, however, the possible intermolecular association may be screened due to participation of the boronic acid group in the intra-molecular hydrogen bonding formation. The PSNB2-based dynamic aggregates as discovered were further studied for the sensing of saccharides, such as fructose. It was demonstrated that the excimer emission of the system is sensitive to the presence of the saccharide, and the response is instantaneous. The DL of the test could be lower than 10 μM. Interestingly, the adduct of PSNB2 with fructose, PSNB2-F, could be further used as an ideal probe of FA in aqueous phase due to dimerization of PSNB2-F in the presence of the analyte. Again, the sensing is sensitive and fast. The DL of this test is also lower than 10 μM. Moreover, the two sensing processes can also be performed in a visualized manner. It is believed that the work presented not only reports on two smart fluorescent sensors for fructose and FA, but also represents examples for how to develop fluorescent sensors via utilization of the principles of dynamic chemistry.

## Experimental Section

### Materials and Methods

Pyrenesulfonyl chloride (PSC) was synthesized by adopting a literature method^60^. Organic liquids used throughout were of analytical grade and used as received, or dried to eliminate any water residue if necessary. Other reagents, excepting those specified, were of analytical grade and used without further purification. Water used in this work was acquired from a Milli-Q reference system.

Steady-state fluorescence measurements were performed at room temperature on a time-correlated single photon counting fluorescence spectrometer (Edinburgh Instruments FLS 920). Time-resolved emission spectra (TRES) were recorded on the same system using EPL-343 picosecond pulsed diode laser as an excitation source. The wavelength range of the TRES measurements was maintained from 370 nm to 652 nm. The monochromator was driven in a 3-nm step under the control of a computer. The accumulation time for each decay curve (a specific *λ*_ex_/*λ*_em_) was 120 s. Temperature-dependent fluorescence measurements were performed with thermo-electrically temperature-controlled cuvette holder and the temperature was controlled with a Quantum Northwest TC 125 temperature controller.

For ^1^H NMR study, the sample containing the as-obtained compound and DMSO was prepared in a NMR tube, and the chemical shifts were determined by using a Fourier digital NMR spectrometer (Bruker Avance, 600 MHz). In addition, diffusion ordered NMR spectroscopy was performed by using the Fourier digital NMR spectrometer.

The MS data were collected on a Bruker maxis UHR-TOF mass spectrometer in ESI positive mode.

Transmission electron microscopy (TEM) images were obtained using Tecnai G2 F20 field transmission electron microscope at an acceleration voltage of 200 kV.

Dynamic light scattering (DLS) measurements were performed on a Malvern Zetasizer Nano-ZS90.

The XRD patterns of the solid powdered compound of PSNB1 and PSNB2 were collected by a Rigalcu D/Max-3c diffractometer with Cu Kα radiation (λ = 0.154 nm). The tube voltage and amperage were set at 40 kV and 30 mA respectively. PSNB1 and PSNB2 were scanned from 2° to 50° (2θ) with a step size of 0.02°, respectively, and the scan rate was 5°/min.

### Sample Preparations

#### Preparation of Stock Solution of PSNB Derivatives in Methanol

The stock solutions of PSNB derivatives with a concentration of 1 × 10^−2^ M were prepared by dissolving certain amount of the PSNB derivatives in 10 mL methanol. The solutions as prepared were stored in a refrigerator under 8 °C before use.

#### Preparation of pH Titration Sample

To an aqueous solution of NaCl (100 mM) was add fructose and methanol stock solution of PSNB2, then the solution, which contains 1% (v/v) methanol, was titrated with NaOH and HCl. In this way, samples with different pH but same volume (1 mL) were prepared and transferred, separately, to vials for aging. The aging was lasted for 3 h before fluorescence measurements were conducted.

#### Preparation of Sample for TEM Measurements

Samples for TEM measurements were prepared by immerging a pure carbon-coated copper grid into water solution of PSNB2 (0.1 mM), then the copper grid was removed out from the solution, and then the solvent on its surface was evaporated completely at room temperature.

## Additional Information

**How to cite this article**: Chang, X. *et al*. Dynamic Covalent Chemistry-based Sensing: Pyrenyl Derivatives of Phenylboronic Acid for Saccharide and Formaldehyde. *Sci. Rep.* **6**, 31187; doi: 10.1038/srep31187 (2016).

## Supplementary Material

Supplementary Information
