# Supplementary material for: Dynamic Covalent Chemistry-based Sensing: Pyrenyl Derivatives of Phenylboronic Acid for Saccharide and Formaldehyde
Source: Sci Rep. 2016 Aug 8;6:31187. doi: 10.1038/srep31187 (PMC4976341; doi:10.1038/srep31187)
Supplement: Supplementary Information [file srep31187-s1.doc]

**Electronic Supplementary Information**

***for***

**Dynamic Covalent Chemistry-based Sensing: Pyrenyl Derivatives of Phenylboronic Acid for Saccharide and Formaldehyde**

Xingmao Chang^1^, Jiayun Fan^2^, Min Wang^2^, Zhaolong Wang^2^, Haonan Peng^2^, Gang He^3^, Yu Fang^2,*^

^1^Key Laboratory of Applied Surface and Colloid Chemistry (Ministry of Education), Shaanxi Normal University, School of Materials Science and Engineering, Xi'an, 710119, P. R. China

^2^Key Laboratory of Applied Surface and Colloid Chemistry (Ministry of Education), Shaanxi Normal University, School of Chemistry and Chemical Engineering, Xi'an, 710119, P. R. China

^3^Frontier Institute of Science and Technology, Xi'an Jiaotong University, Xi’an 710119, P. R. China

1. **Synthesis of PSNB1, PSNB2 and PSNP**

The PSNB derivatives used in this work were synthesized in the procedures schematically shown in Scheme S1. The details are given below.

§


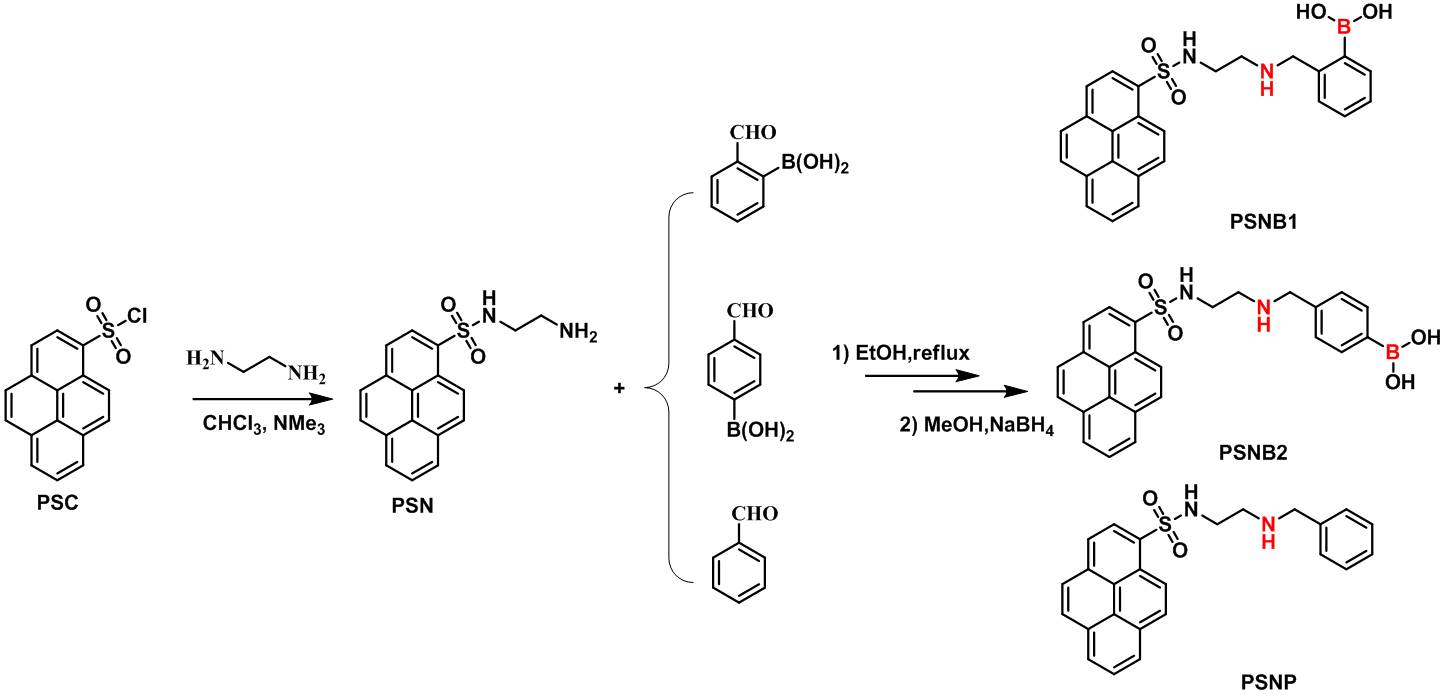


**Scheme S1** Synthesis routes of compounds PSNB1, PSNB2 and PSNP

*Preparation of PSN*: PSN was prepared by the method described in one of our earlier publications.^S1^ The ^1^H NMR data (δ ppm, 600 MHz, DMSO-d6) of the compound are as follows: 9.01-8.99(1H), 8.61-8.59(1H), 8.50-8.38(5H), 8.31-8.29(1H), 8.24-8.20(1H), 2.81-2.78(2H) and 2.44-2.41(2H).

*Preparation of PSNB1*: A certain amount of PSN (0.32 g, 1 mmol) was dissolved in 20 mL of absolute ethanol. After addition of 2-formylphenylboronic acid (0.15 g, 1mmol), the reaction mixture was refluxed in an air bath under argon atmosphere for 6 h, then, the solution was cooled and allowed to stand at room temperature overnight, and then ethanol was evaporated completely under reduced pressure. The imine intermediate as obtained was dissolved in 15 mL of absolute methanol. Sodium borohydride was then added slowly with stirring at room temperature for 4 h. The volatile components were removed on a rotary evaporator. The crude product as obtained was purified on a silica-gel column using a mixture of CH_2_Cl_2_/methanol (from 10:1 to 1:5 by volume) as eluent. The purified PSNB1 as obtained was collected as yellowish solid; ^1^H NMR (δ ppm, 600 MHz, DMSO-d6 with 20 μL of DCl): 8.98-8.97(1H), 8.62-8.61(1H), 8.52-8.41(5H), 8.32-8.31(1H), 8.25-8.22(1H), 7.78-7.76(1H), 7.40-7.33(3H), 4.26(2H) and 2.98-2.96(2H). MS (m/z): calculated [(M + H)^+^]: 459.1548, found: 459.1549.

*Preparation of PSNB2*: PSNB2 was prepared in a similar way by using 4-formylphenylboronic acid instead of 2-formylphenylboronic acid. A yellowish solid was obtained, and its characterization data are given here. ^1^H NMR (δ ppm, 600 MHz, DMSO-d6 with 20 μL of DCl): 8.98-8.97(1H), 8.62-8.61(1H), 8.52-8.41(5H), 8.32-8.31(1H), 8.25-8.22(1H), 7.79-7.78(2H), 7.44-7.43(3H), 4.09(2H), 3.32-3.18(2H) and 2.99-2.97(2H). MS (m/z): calculated [(M+CH_3_OH-H_2_O+H)^+^]: 473.1305, found: 473.1315 and calculated [(M+2CH_3_OH-2H_2_O+H)^+^]: 487.1862, found: 487.1870.

*Preparation of PSNP*: PSNP was prepared in a similar way by using benzaldehyde instead of 2-formylphenylboronic acid. A yellowish solid was obtained, and its characterization data are given here. ^1^H NMR (δ ppm, 600 MHz, DMSO-d6): 8.99-8.97(1H), 8.62-8.60(1H), 8.50-8.40(5H), 8.32-8.30(1H), 8.24-8.22(1H), 7.31-7.30(1H), 7.21-7.15(4H), 3.66(2H), 3.05-2.92(2H) and 2.66-2.64(2H).

1. **Diffusion coefficients of PSNB2 and its control compounds in DMSO-d6** **(Fig. S1)**


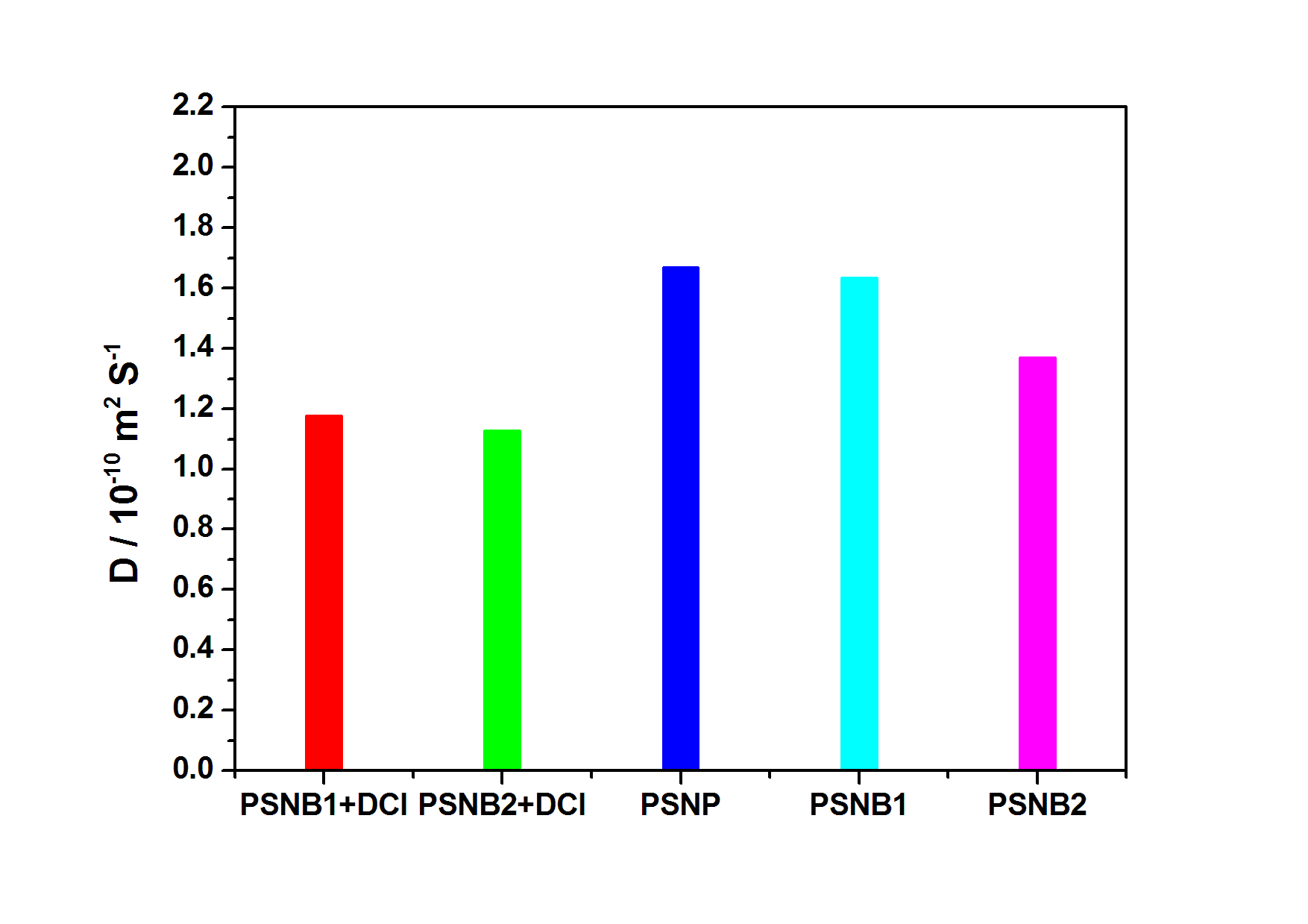


**Fig. S1** Diffusion coefficients D of PSNB1+DCl, PSNB2+DCl, PSNP, PSNB1 and PSNB2 recorded at 298 K in DMSO-d6 (*w/v* = 1.6%). It is believed that protonation of the two compounds must make them exist in ionic associate states, which blocks the hydrogen bonding, and thereby disrupts the possible polymer and the intra-molecular functional group interaction. Moreover, protonation must also reduce the mobility of the molecules of the compounds due to their ionic nature.

1. **XRD studies (Fig. S2)**


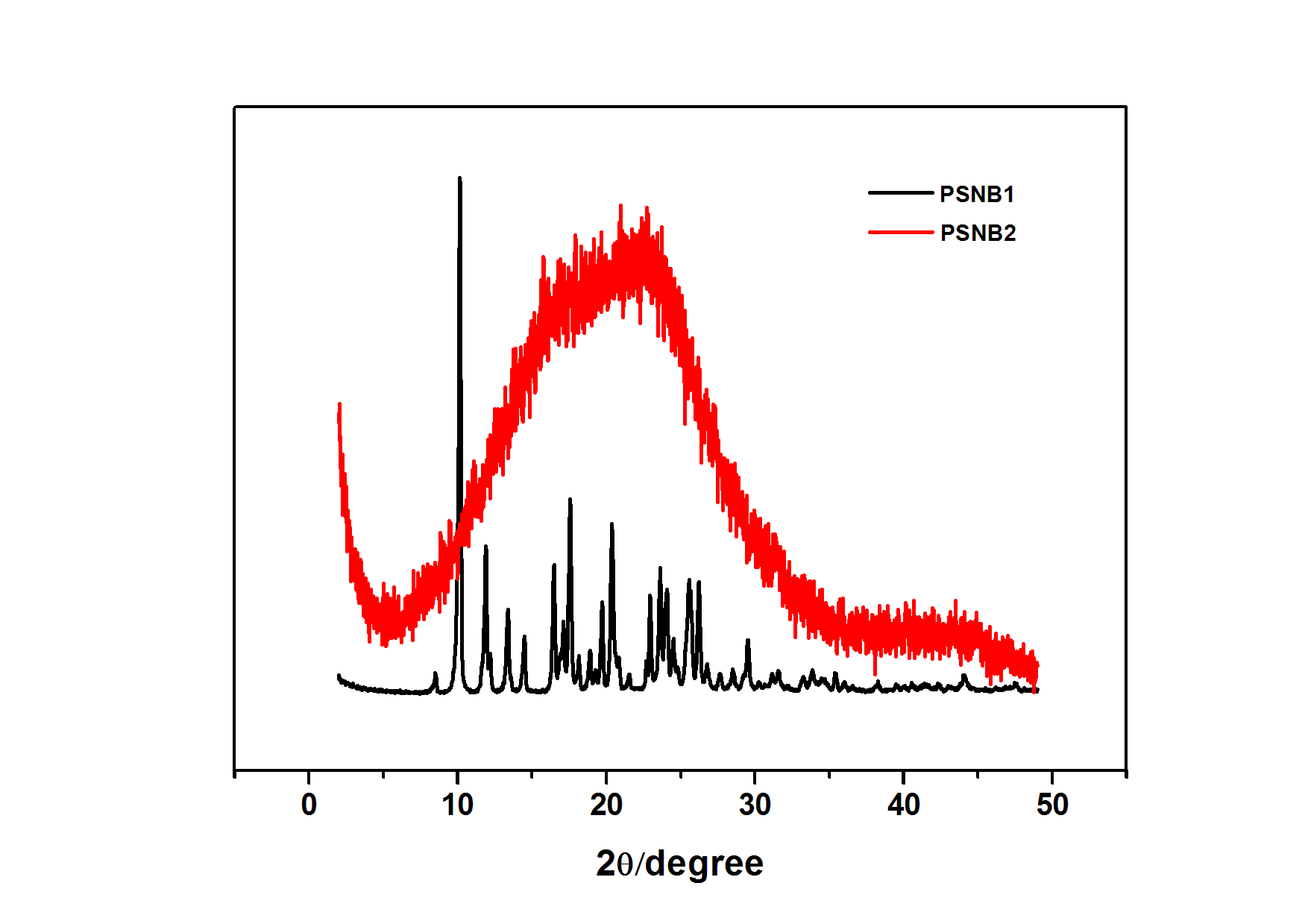


**Fig. S2** Power XRD of PSNB1 and PSNB2

1. **Temperature- and concentration-dependent ^1^H NMR spectra of PSNB2 in DMSO-d6 (****Fig. S3)**


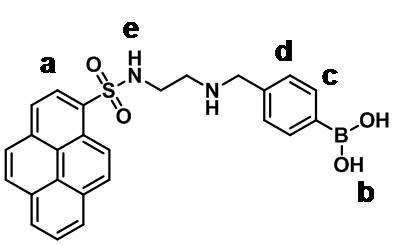

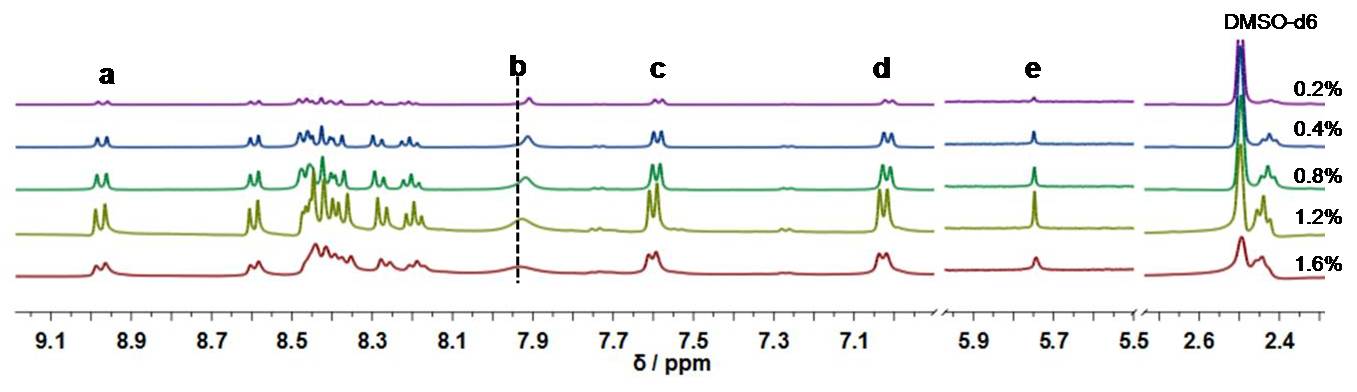


**a)**


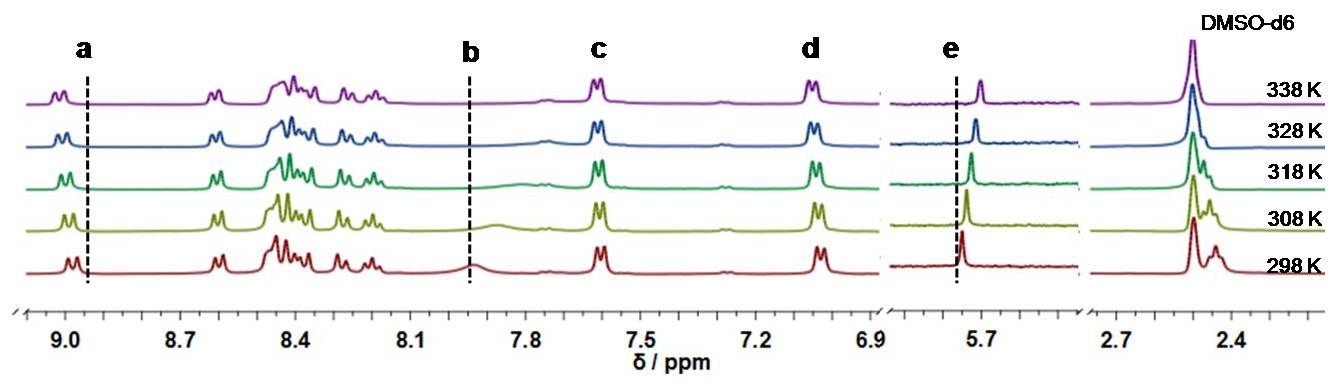


**b)**

**Fig. S3** (a) Temperature-dependent ^1^H NMR spectra of PSNB2 in DMSO-d6 (*w/v* = 1.6%); (b) Concentration-dependent ^1^H NMR spectra of PSNB2 in DMSO-d6 recorded at 298 K.

1. **The TEM images of the PSNB2 aggregates from its aqueous solution (Fig. S4)**


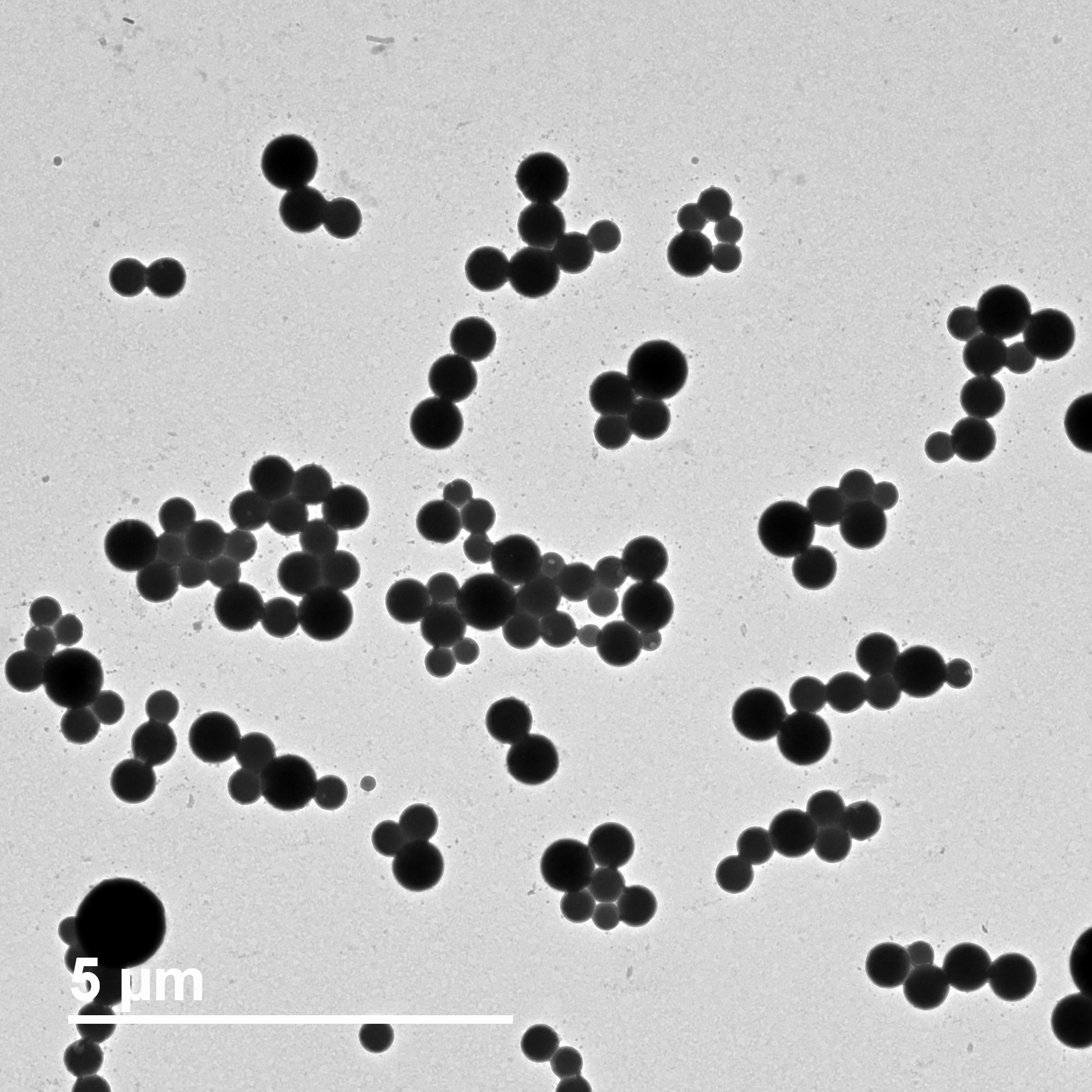


**Fig. S4** TEM images of the aggregates taken from the aqueous solution of PSNB2 (1.0×10^-4^ mol/L) at room temperature.

1. **Plots of I_490_/I_380_ against the pH in the absence and presence of fructose (Fig. S5)**


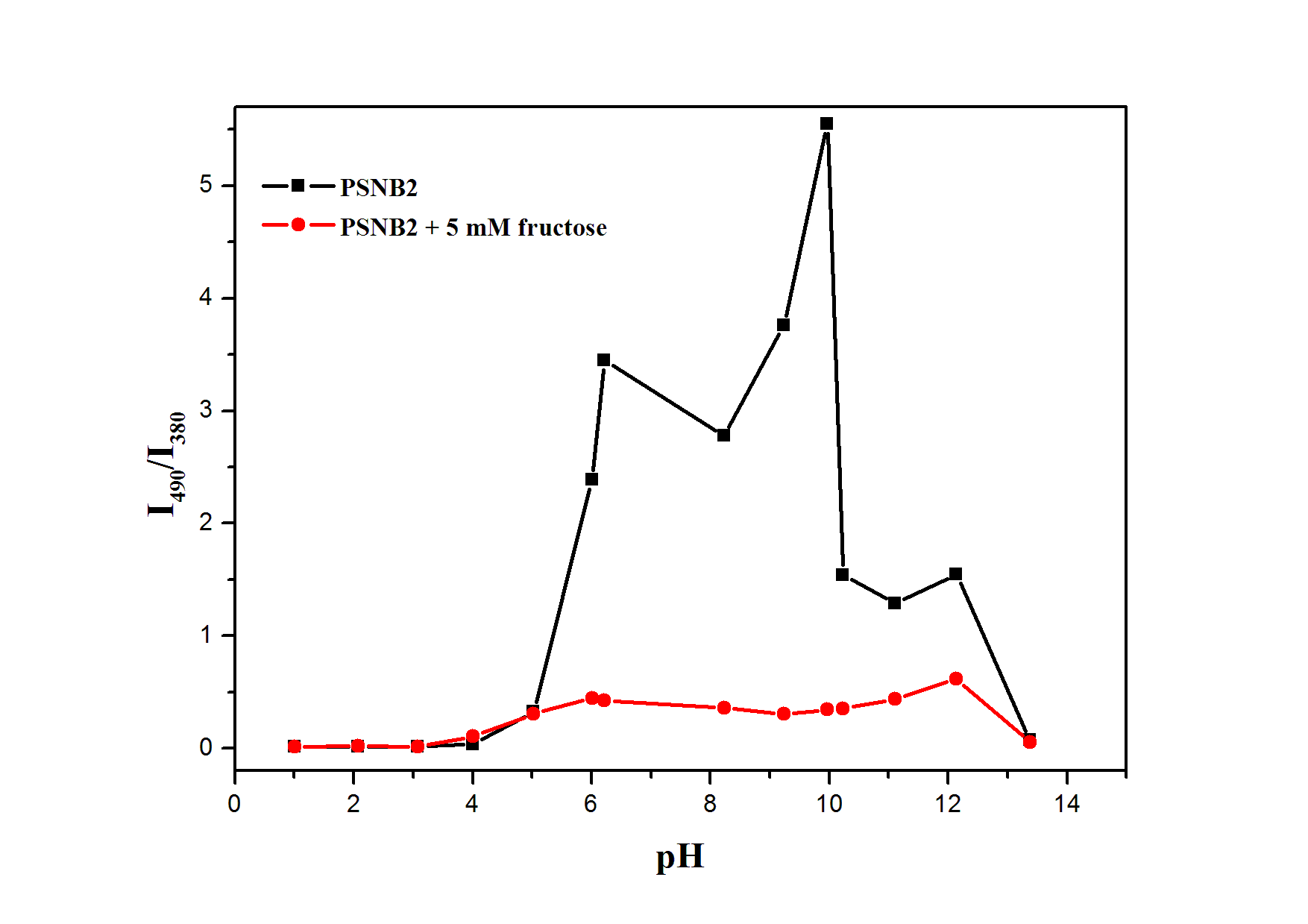


**Fig. S5** Plots of I_490_/I_380_ against the pH in the absence and presence of fructose ([PSNB2]= 1×10^4^ mol/L, [Fructose]= 5 mM)

1. **Fluorescence response of PSNB1 to the fructose (Fig. S6)**


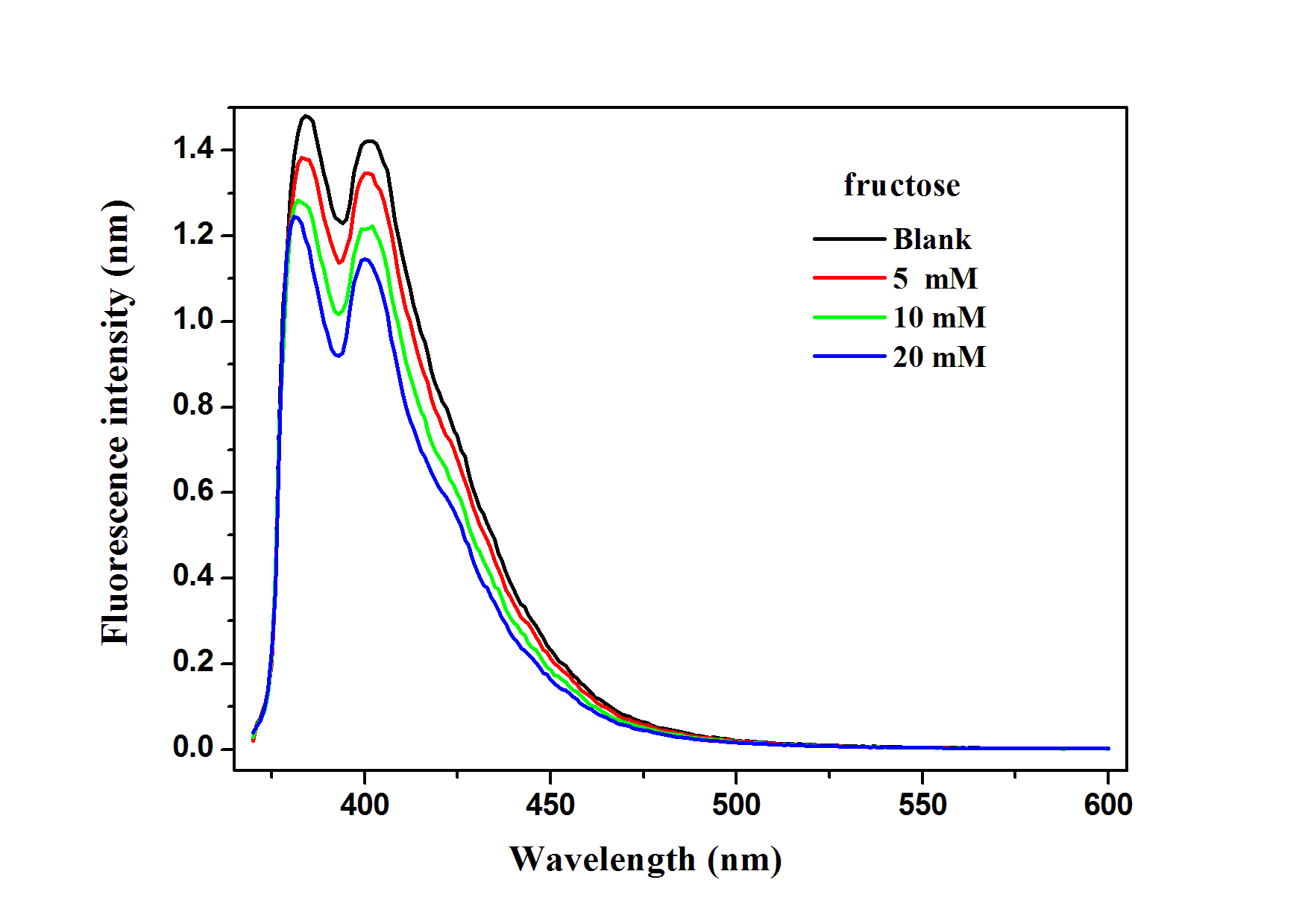


**Fig. S6** Fluorescence emission spectra of PSNB1 in pH 9.0 carbonate buffer containing 1% (v/v) methanol at different concentrations in the presence of fructose.

1. **DLS response of PSNB2 to the fructose** **(Fig. S7)**


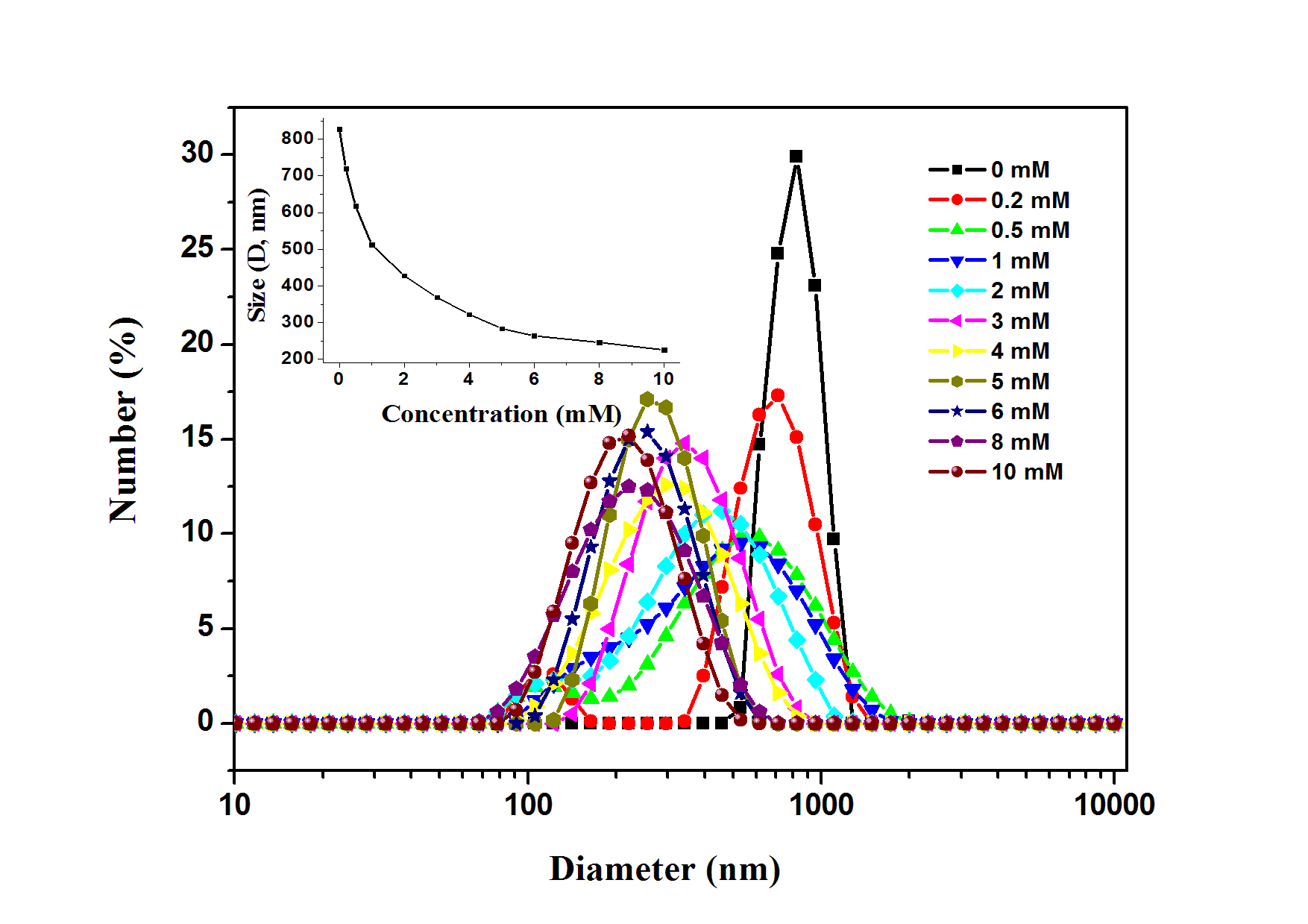


**Fig. S7** Particle size distributions of PSNB2 in aqueous solution (0.1 mM) with different concentrations of fructose, which are the results from dynamic light scattering measurements. Inserted picture is plot of particle size distribution as a function of the concentration of fructose in the aqueous solution of PSNB2.

1. **Fluorescence response of PSNB2 to F over 10 mins (Fig. S8)**


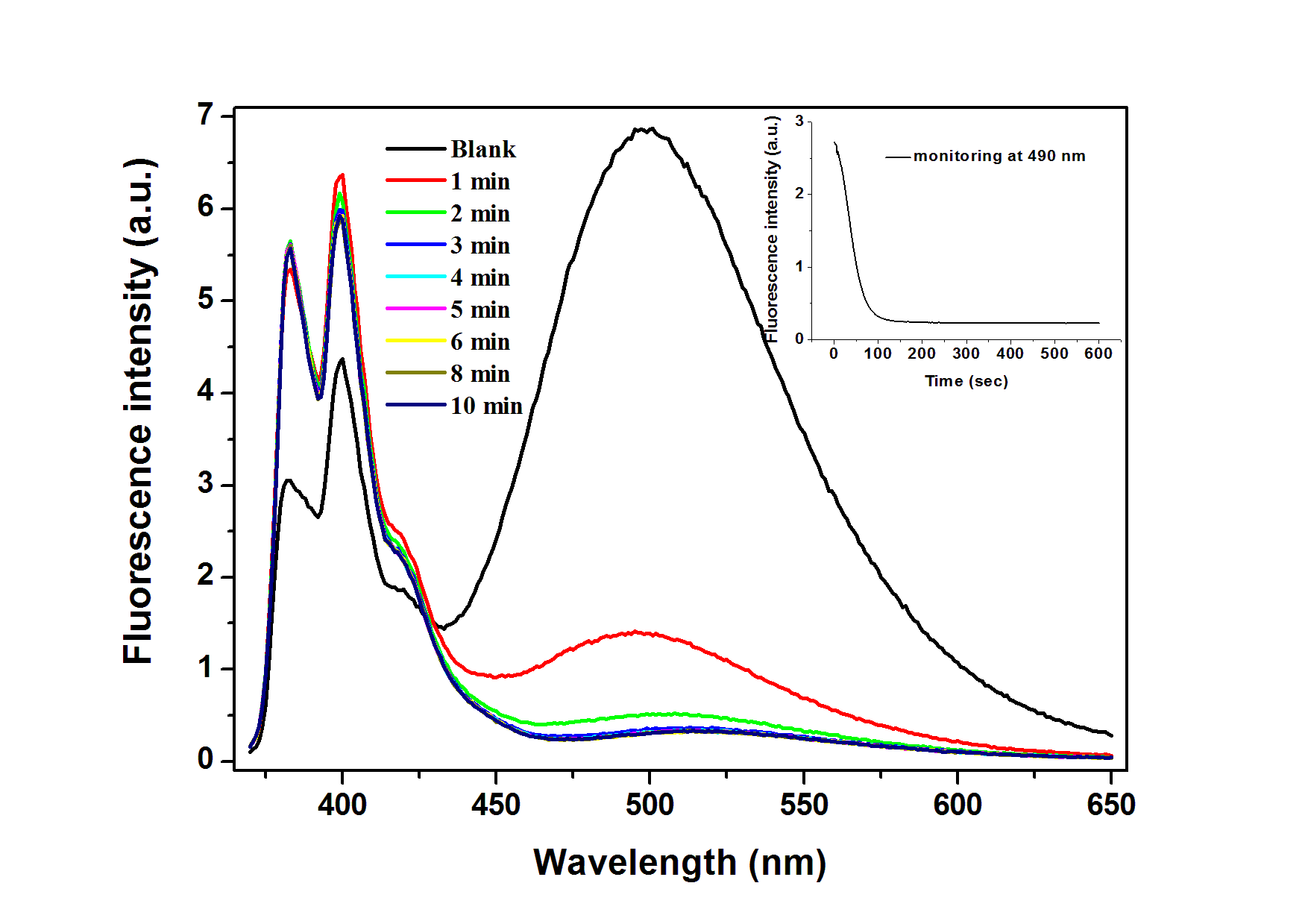


**Fig. S8** Fluorescence response of PSNB2 to 5 mM F over 1-10 mins.

1. **Fluorescence response of PSNB2-F to FA over 10 mins (Fig. S9)**


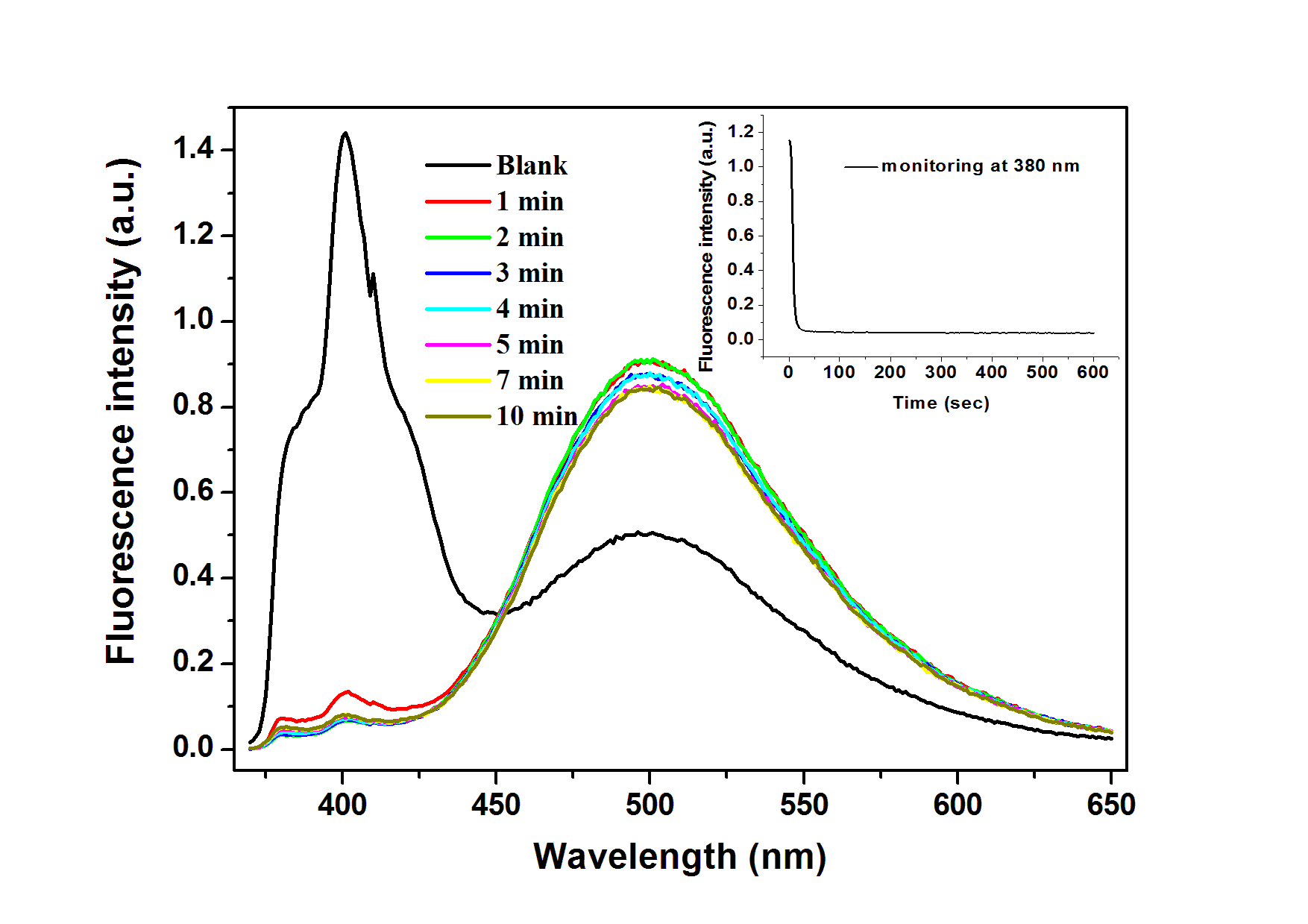


**Fig. S9** Fluorescence response of PSNB2-F to 1 mM FA over 1-10 mins.

1. **MS of the adduct (PSNB2-F-FA) of PSNB2-F and FA (Fig. S10)**

To further identify the structure of adduct of PSNB2-F and FA, FA-PSNB2-F, which was obtained through adding FA into the mixture solution of PSNB2 and fructose (c.f. Scheme 1), high-resolution MS measurements were conducted. The expected signal of PSNB2-F-FA did not appear due to its possible breakage during the ionization process (c.f. Fig. S9a). This is confirmed by the appearances of the peaks at m/z = 603.1957 and m/z = 615.1958, which can be ascribed to A+1 and B-1, respectively, as shown in the Fig. S9b. The assignment was further confirmed by theoretical calculations as depicted in Figs. S9c and 9d, respectively.


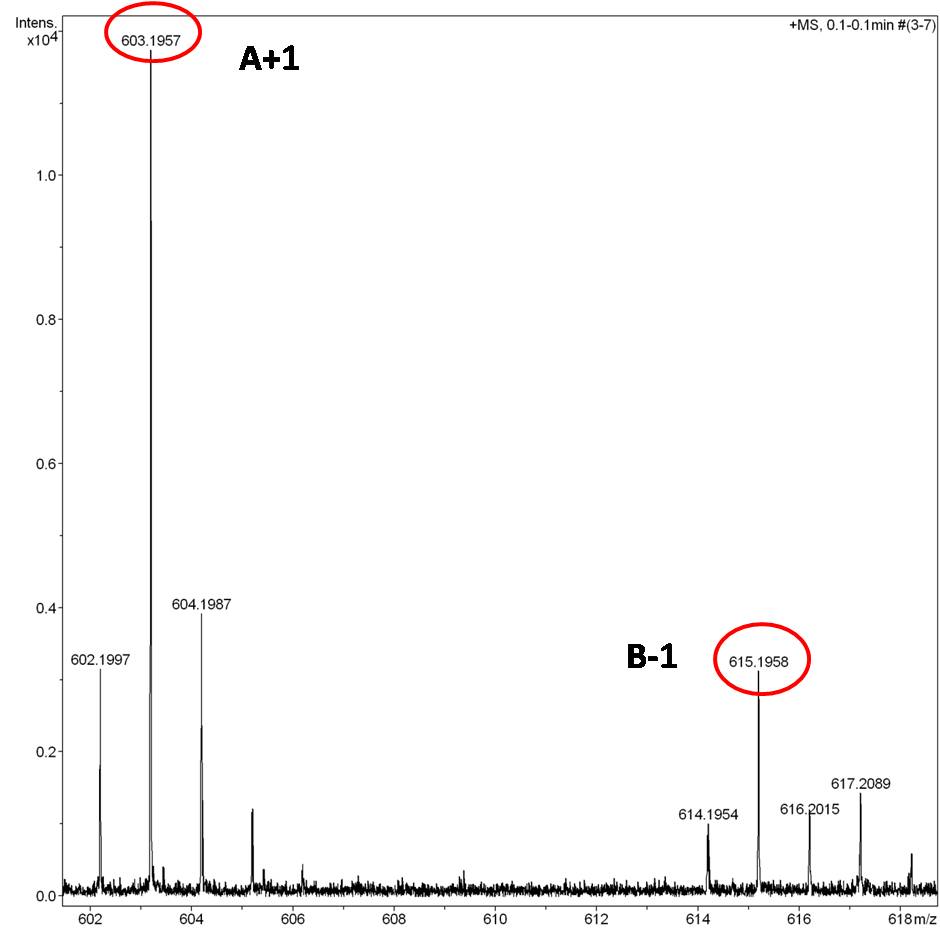


**a)**


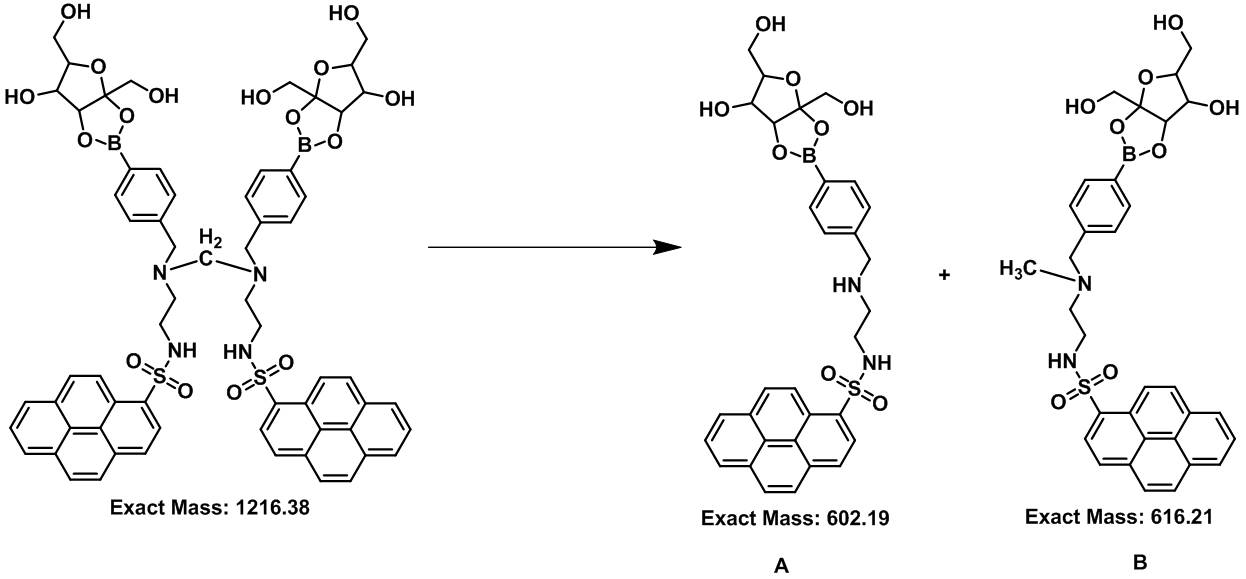


**b)**

**Fig. S10** a) The MS spectra of PSNB2-F-FA; b) The possible fragmentation pattern of PSNB2-F-FA


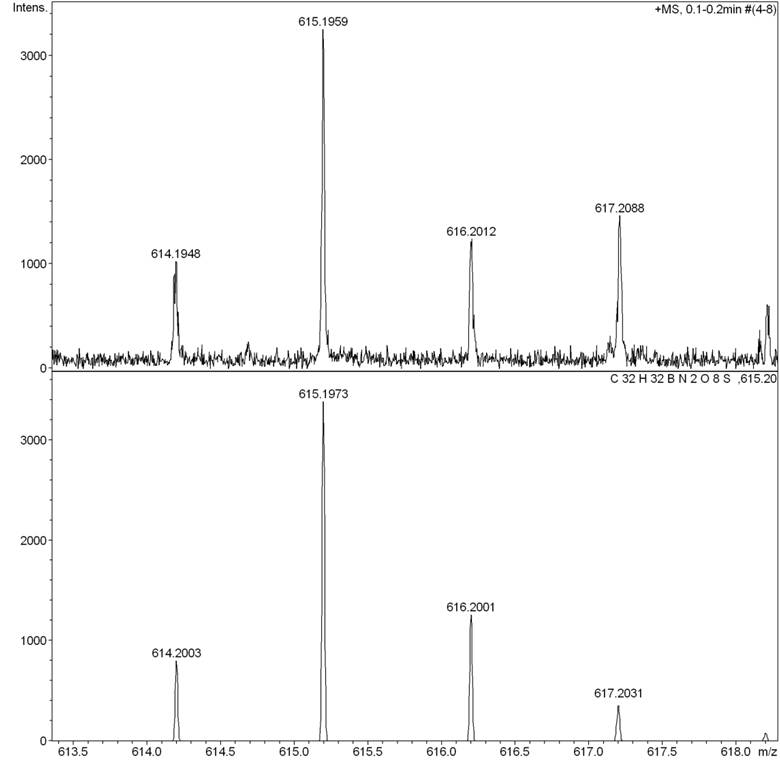

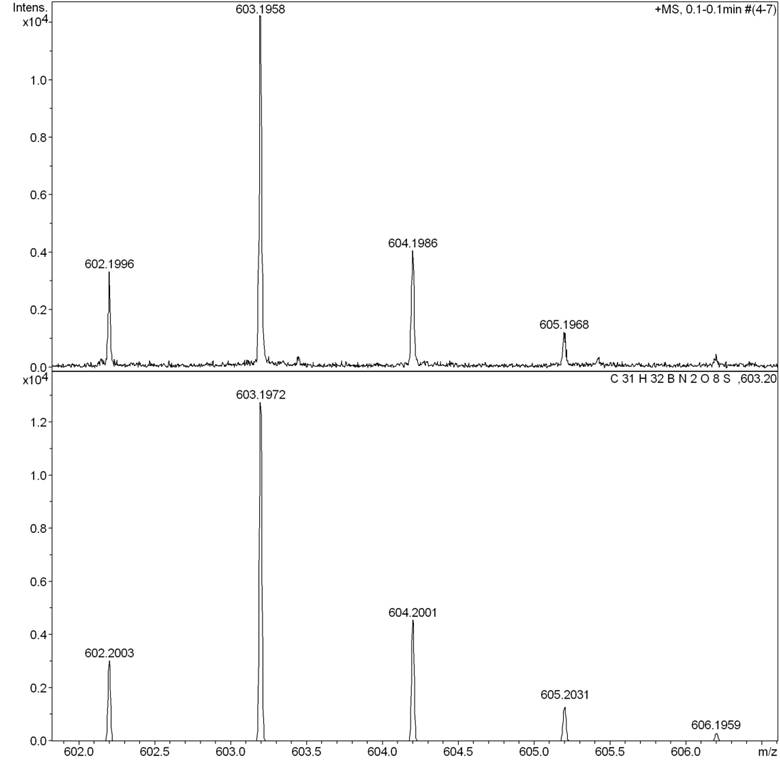


**c)**  **d)**

**Fig. S10** c) The selected MS spectra of PSNB2-F-FA at m/z = 615 with the calculated one; d) The selected MS spectra of PSNB2-F-FA at m/z= 603 with the calculated one.

1. **^1^H NMR spectra of model reaction based on DEA and FA (Fig. S11).**

In order to obtain direct evidence for the reaction between secondary amine and FA, diethylamine (DEA) was employed as an example secondary amine to conduct the reaction. ^1^H NMR spectrum of the mixture of DEA and FA was recorded as a function of time, and the results are shown in Fig. S10. Reference to the traces shown in the Figure reveals that the initial ^1^H NMR spectrum of DEA and FA shows no impurity peaks. However, a few minutes later, there appeared a new resonance at 4.25 ppm, which can be assigned to methylene structure as expected, a direct evidence for the crosslinking.


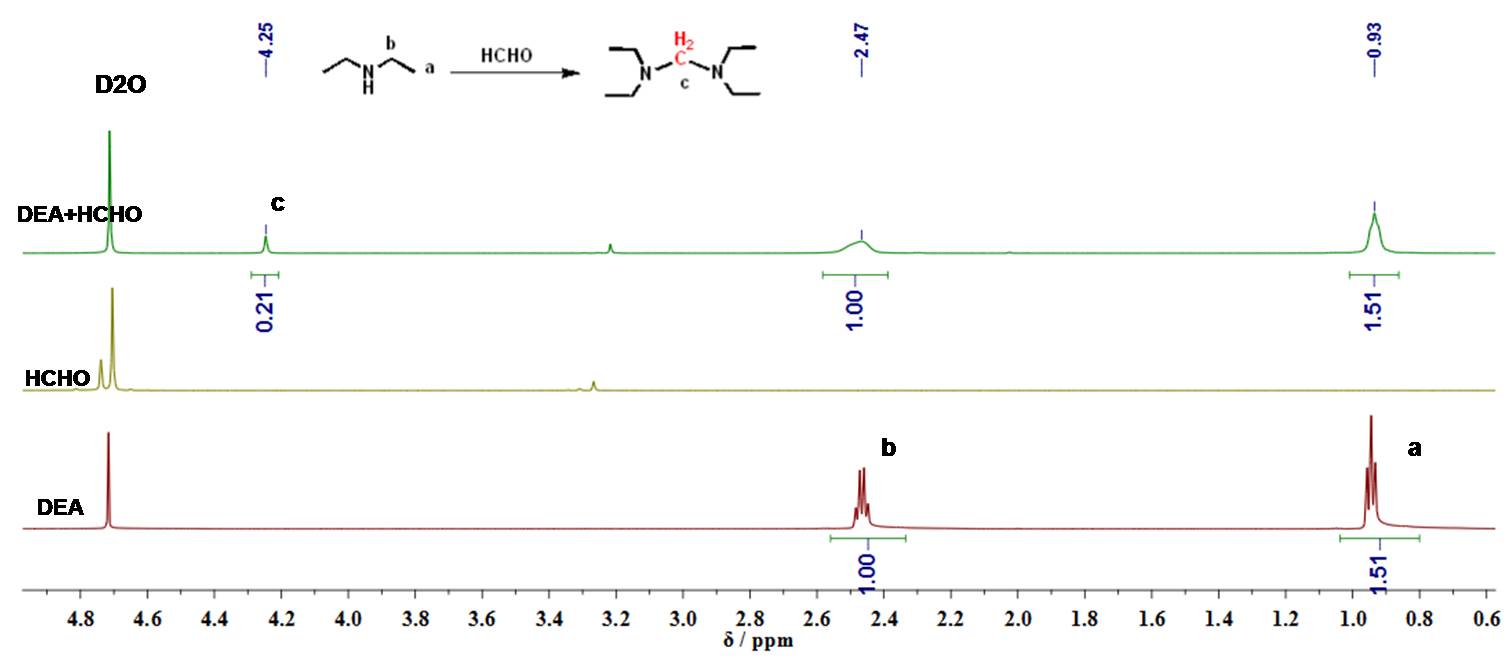


**Fig. S11** ^1^H NMR spectra of model reaction with DEA and HCHO in D_2_O-d2.

1. **Figures for characterizations of PSNB1, PSNB2 and PSNP (Figs. S12-S17)**


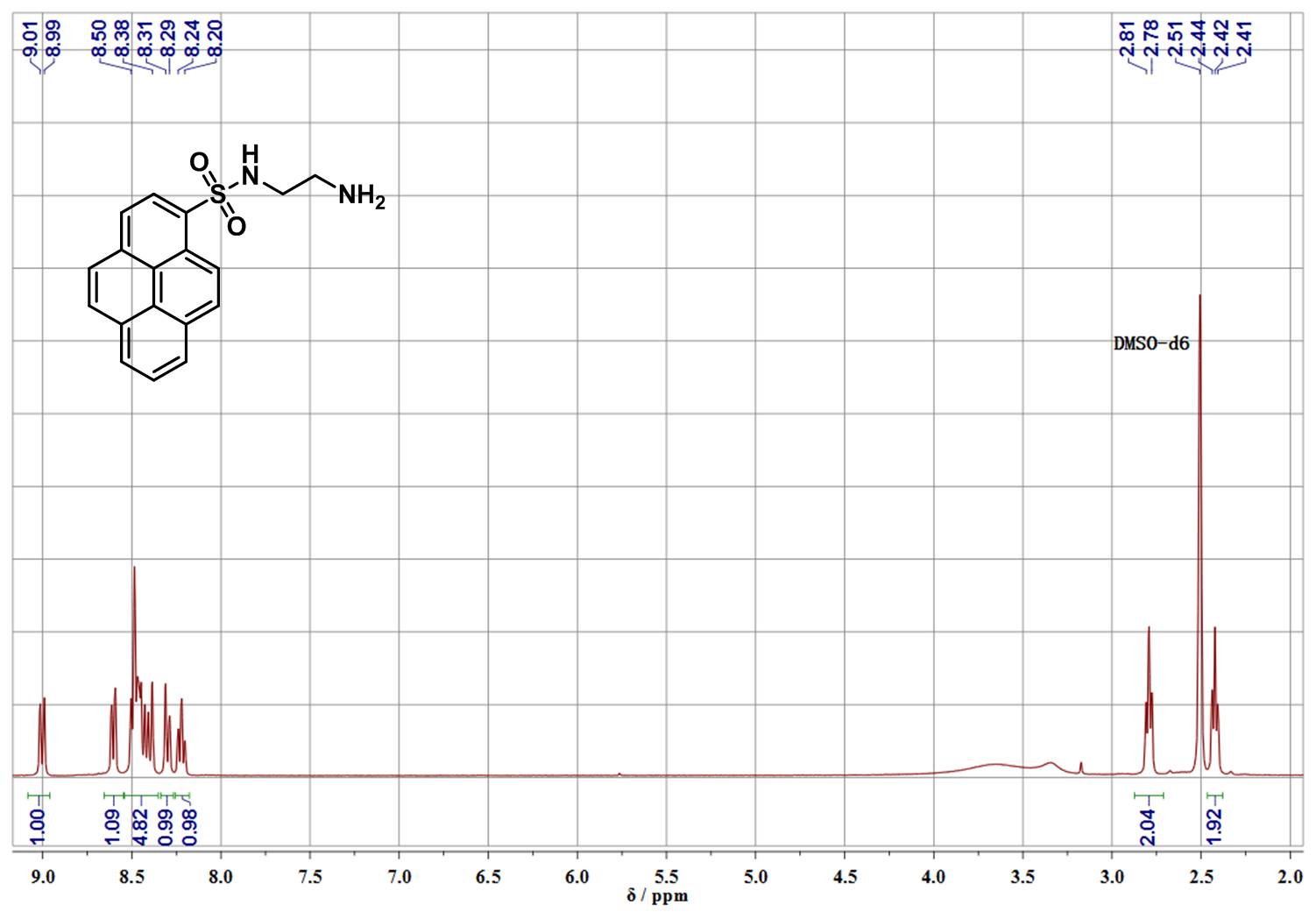


**Fig. S12** ^1^H NMR spectra of PSN in DMSO-d6


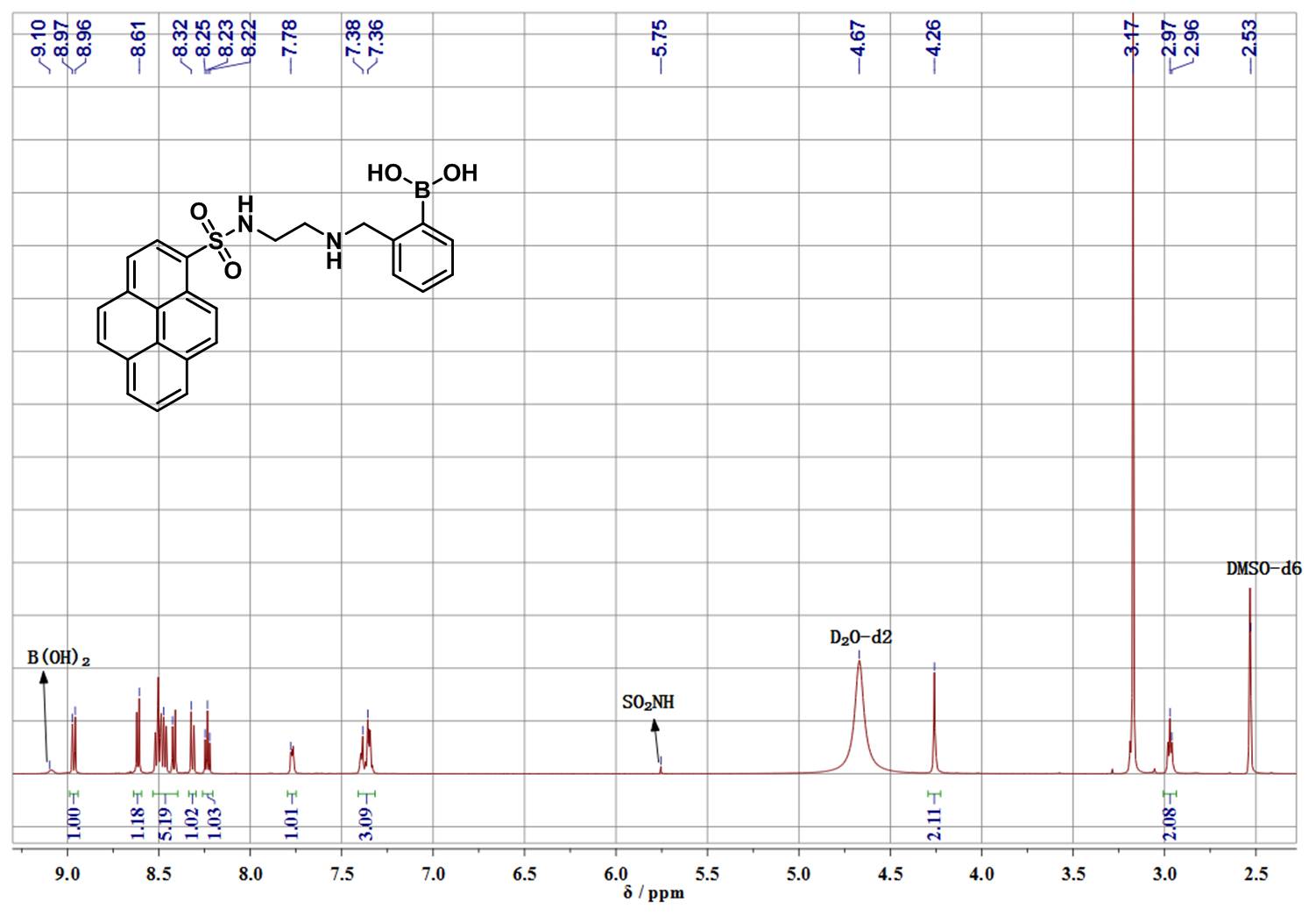


**Fig. S13** ^1^H NMR spectra of PSNB1 in DMSO-d6 with 20 μL of DCl


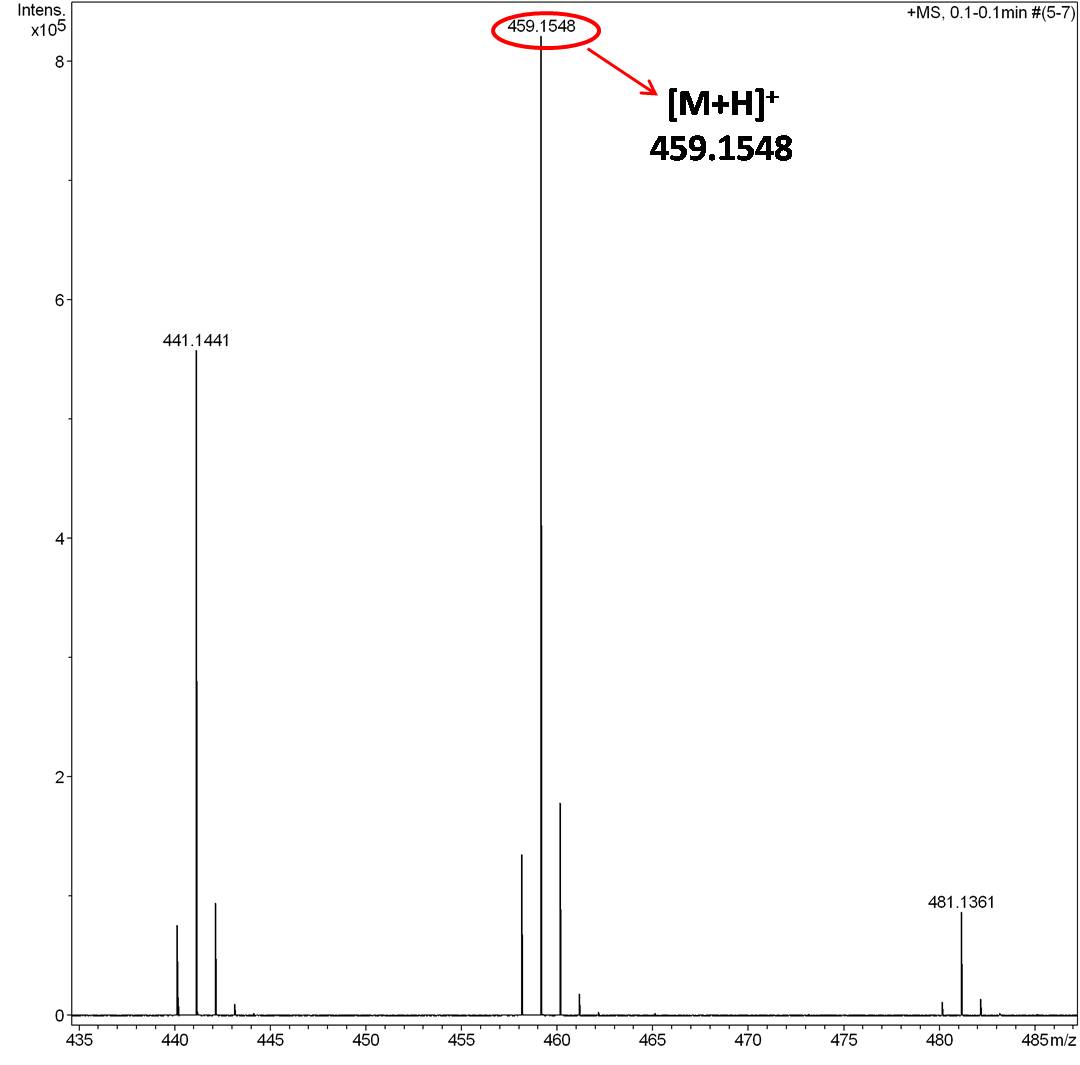

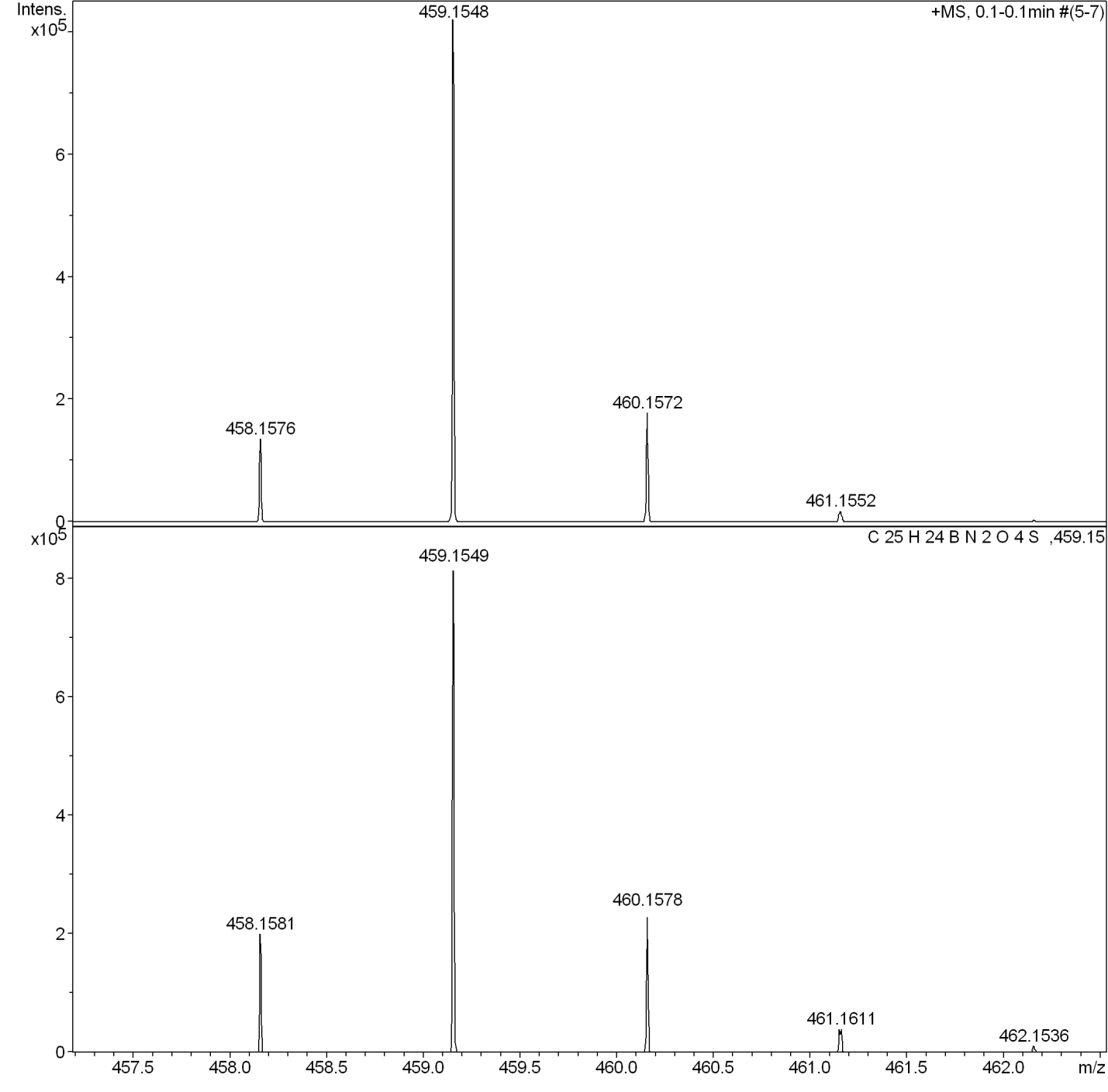


**a) b)**

**Fig. S14** a) The MS spectra of PSNB1; b) The selected MS spectra of PSNB1 at m/z= 459 with the calculated one


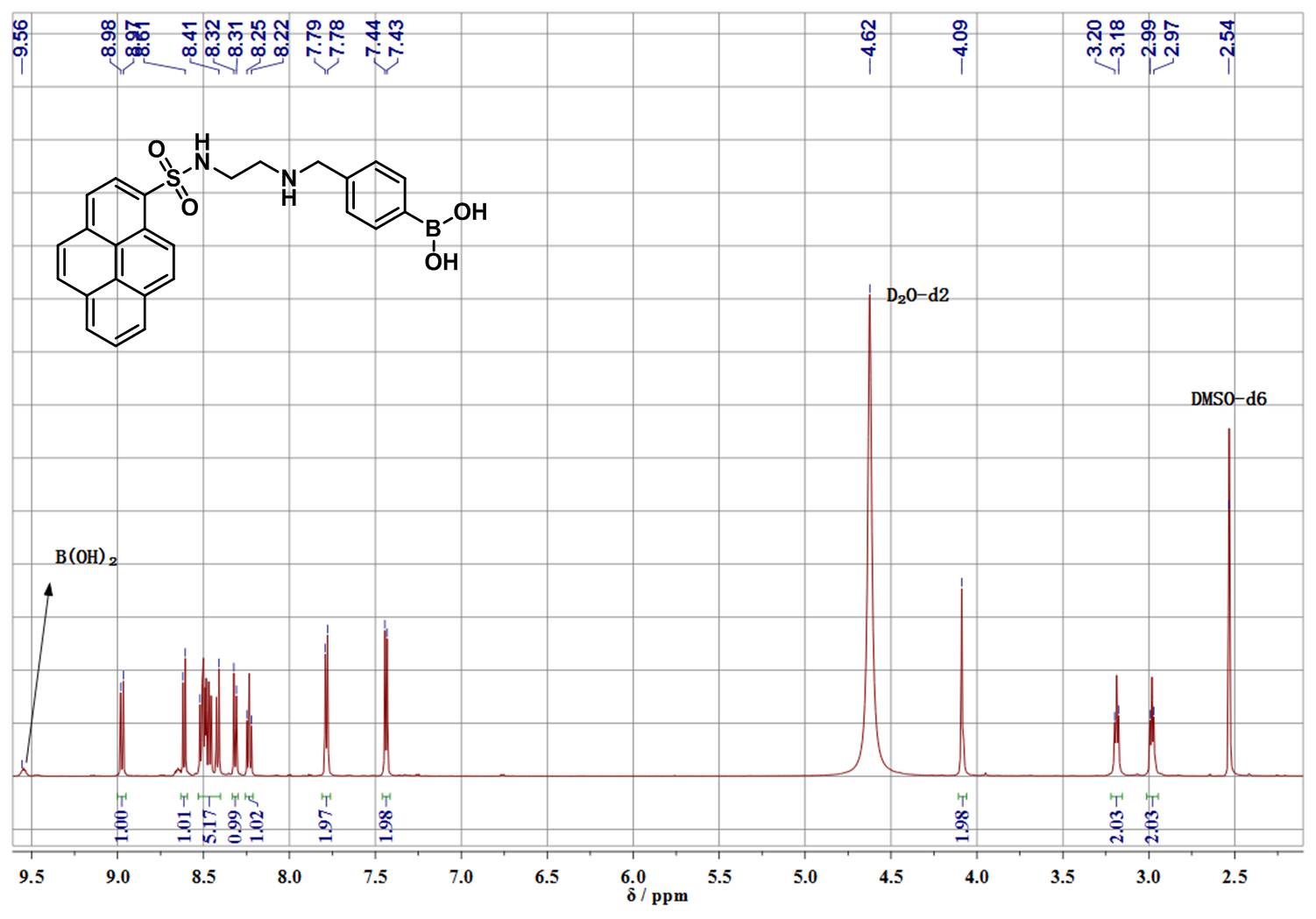


**Fig. S15** ^1^H NMR spectra of PSNB2 in DMSO-d6 with 20 μL of DCl


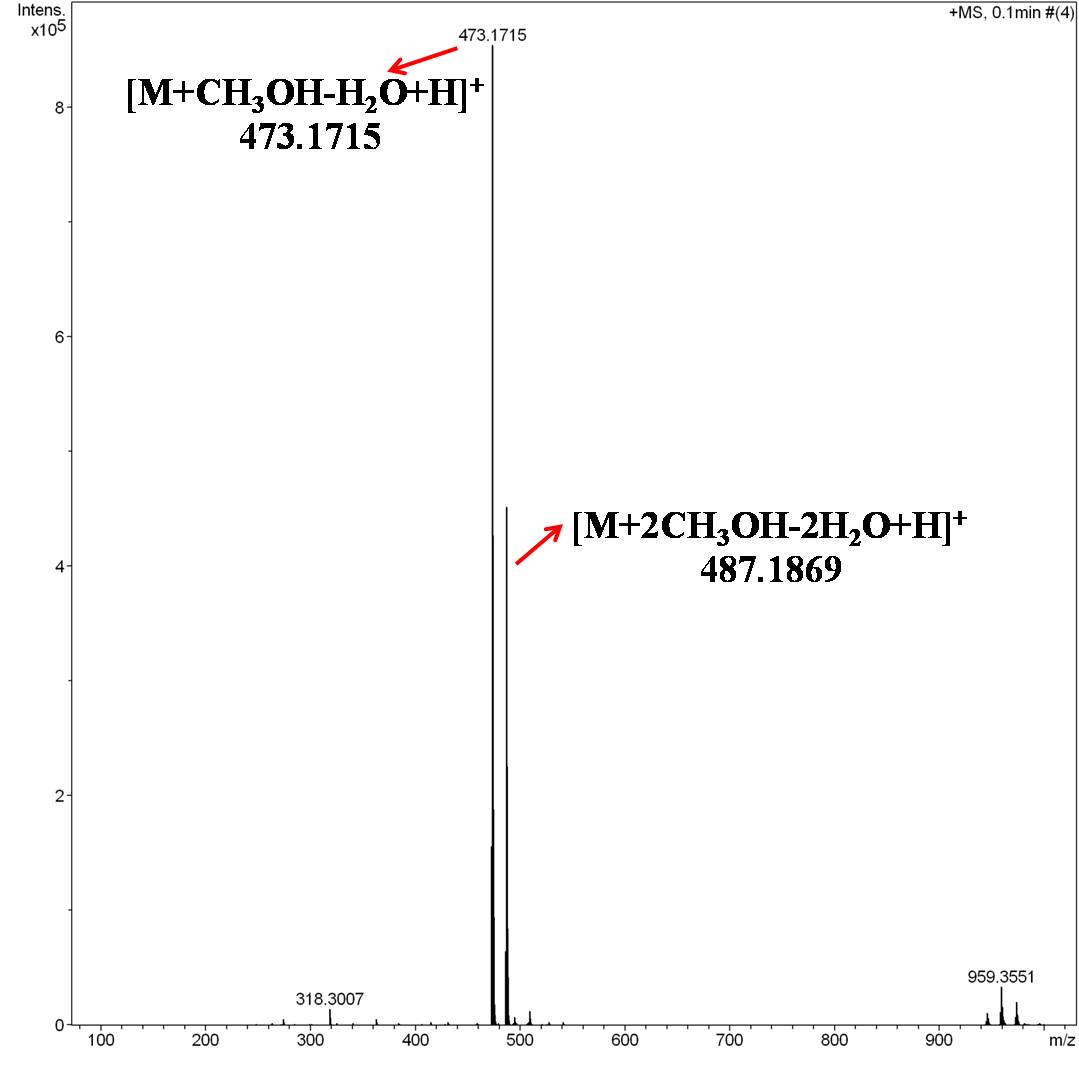

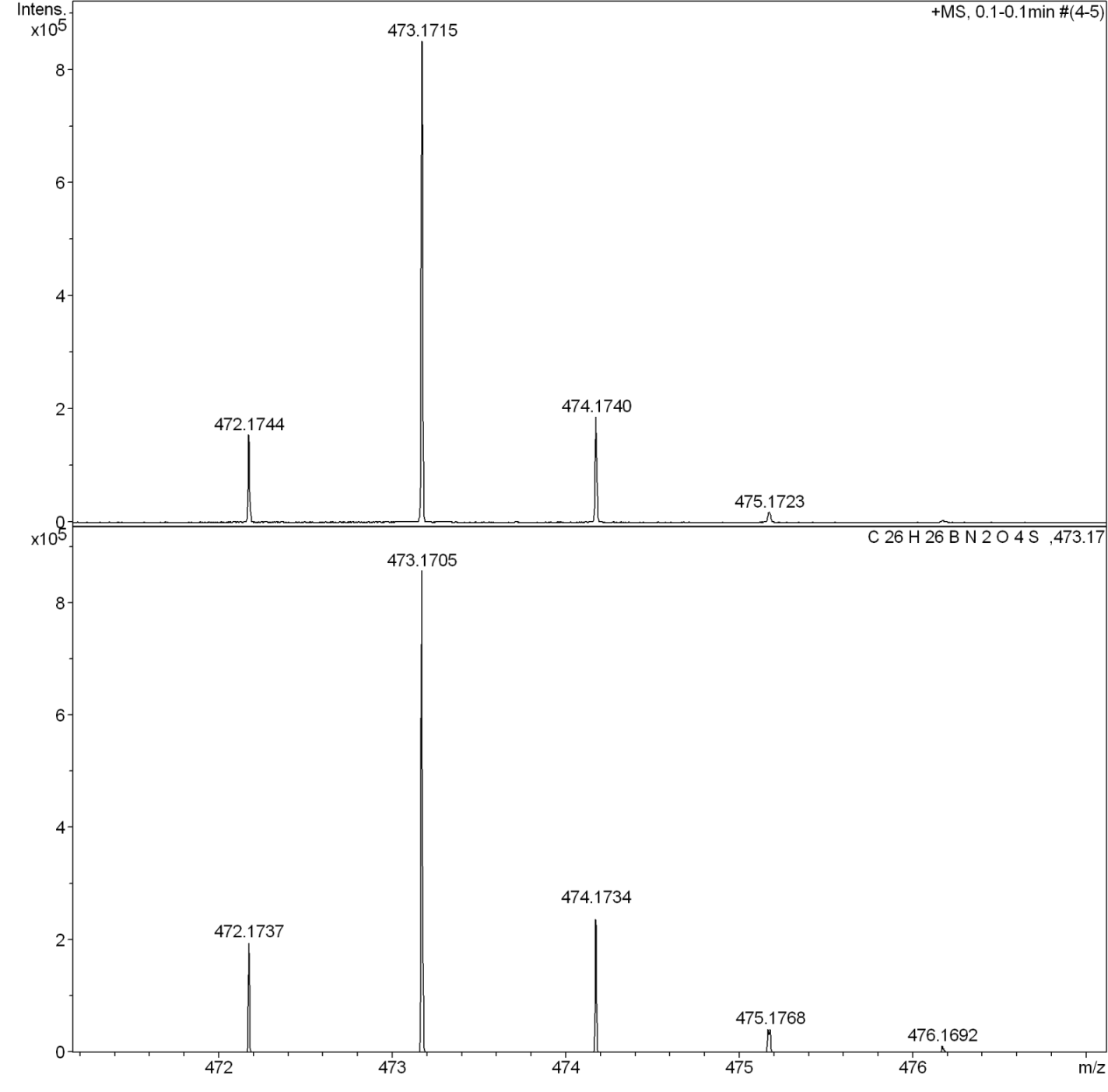


**a) b)**

**
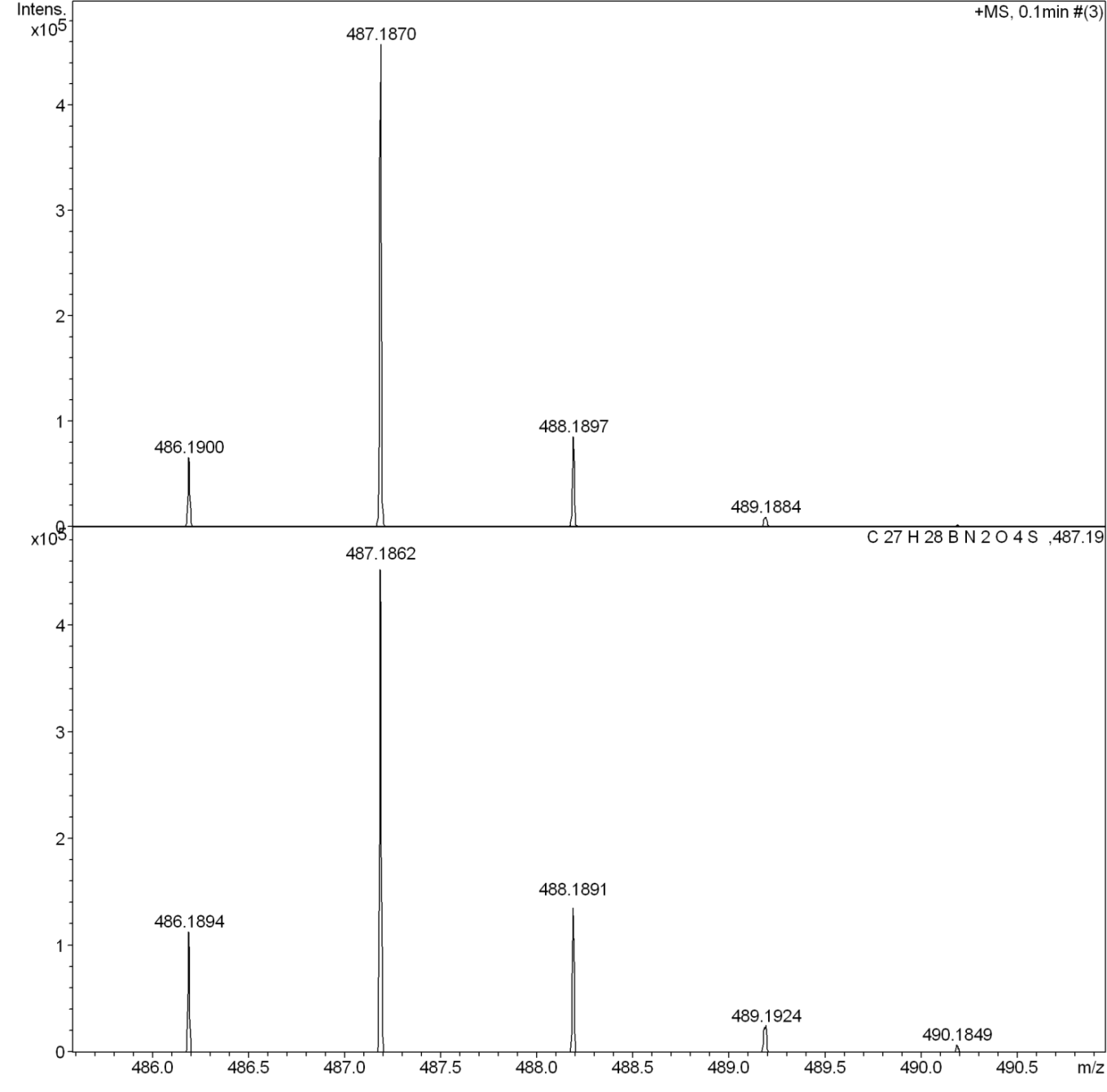
**

**c)**

**Fig. S16** a) The MS spectra of PSNB2; b) The selected MS spectra of PSNB2 at m/z= 473 with the calculated one; c) The selected MS spectra of PSNB2 at m/z= 487 with the calculated one.


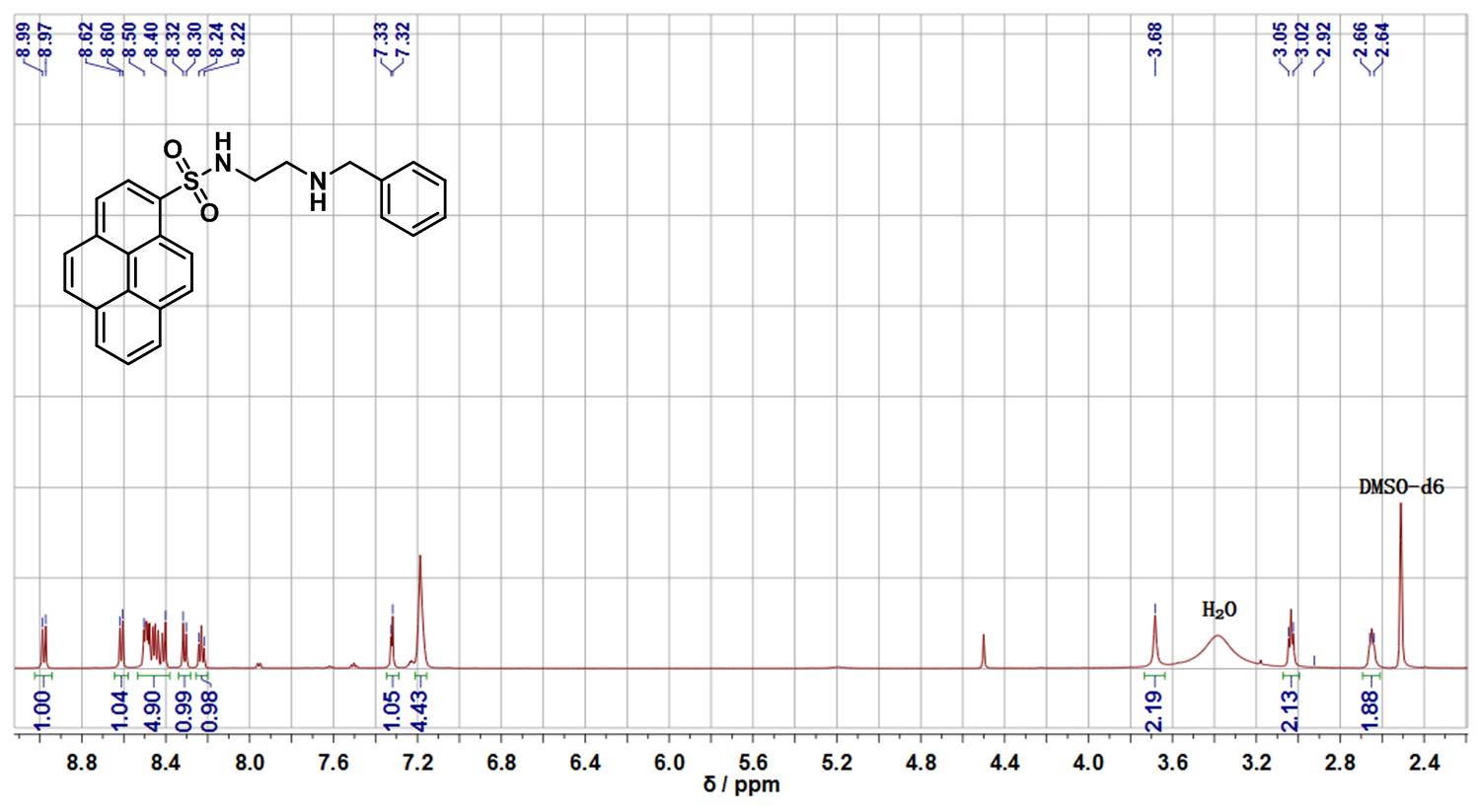


**Fig. S17** ^1^H NMR spectra of PSNP in DMSO-d6

**Reference**

S1. L. Gao, Y. Fang, F. Lü, M. Cao, L. Ding. *Appl. Surf. Sci.*, 2006, **252**, 3884-3893.
